# Spatial expression of transcription factors in *Drosophila* embryonic organ development

**DOI:** 10.1186/gb-2013-14-12-r140

**Published:** 2013-12-20

**Authors:** Ann S Hammonds, Christopher A Bristow, William W Fisher, Richard Weiszmann, Siqi Wu, Volker Hartenstein, Manolis Kellis, Bin Yu, Erwin Frise, Susan E Celniker

**Affiliations:** 1 Department of Genome Dynamics, Division of Life Sciences, Lawrence Berkeley National Laboratory, Berkeley, CA 94720, USA; 2 Computer Science and Artificial Intelligence Laboratory, Massachusetts Institute of Technology, Cambridge, MA 02139, USA; 3 The Broad Institute of Massachusetts Institute of Technology and Harvard, Cambridge, MA 02142, USA; 4 Current Address: Institute for Applied Cancer Science, The University of Texas MD Anderson Cancer Center, Houston, TX 77030, USA; 5 Department of Statistics, University of California Berkeley, Berkeley, CA 94720, USA; 6 Department of Molecular Cell and Developmental Biology, University of California Los Angeles, Los Angeles, CA 90095, USA

## Abstract

**Background:**

Site-specific transcription factors (TFs) bind DNA regulatory elements to control expression of target genes, forming the core of gene regulatory networks. Despite decades of research, most studies focus on only a small number of TFs and the roles of many remain unknown.

**Results:**

We present a systematic characterization of spatiotemporal gene expression patterns for all known or predicted *Drosophila* TFs throughout embryogenesis, the first such comprehensive study for any metazoan animal. We generated RNA expression patterns for all 708 TFs by *in situ* hybridization, annotated the patterns using an anatomical controlled vocabulary, and analyzed TF expression in the context of organ system development. Nearly all TFs are expressed during embryogenesis and more than half are specifically expressed in the central nervous system. Compared to other genes, TFs are enriched early in the development of most organ systems, and throughout the development of the nervous system. Of the 535 TFs with spatially restricted expression, 79% are dynamically expressed in multiple organ systems while 21% show single-organ specificity. Of those expressed in multiple organ systems, 77 TFs are restricted to a single organ system either early or late in development. Expression patterns for 354 TFs are characterized for the first time in this study.

**Conclusions:**

We produced a reference TF dataset for the investigation of gene regulatory networks in embryogenesis, and gained insight into the expression dynamics of the full complement of TFs controlling the development of each organ system.

## Background

Tissue development and organ morphogenesis programs require tight spatial and temporal regulation of gene expression. Gene regulatory mechanisms rely on a wide range of proteins, including transcription factors, co-activators, chromatin remodelers, and signaling pathway components. Site-specific DNA-binding transcription factors (TFs) play a central role in controlling gene expression by interacting with specific regulatory DNA elements to activate or repress transcription. Modulated through signaling pathways and protein interactions, TFs set up the transcriptional programs that ultimately specify cell fates and coordinate tissue and organ formation.

Our understanding of regulatory circuit architecture developed from extensive genetic studies, but most of these focused on a small subset of regulators and examined a limited number of tissues or stages (for example, [1]). Genomic technologies enable systematic annotation of regulatory elements in multiple contexts, and increased the scale of known regulatory connections. Regulatory element annotation efforts are underway for multiple organisms: human [2], fly [3,4], worm [5], and mouse [6], and these datasets provide a foundation to analyze and model gene regulation. However, understanding the spatial and temporal control of gene expression requires the integration of data beyond the catalog of DNA elements. A systematic analysis of TFs requires knowledge of where TFs are expressed, how they interact with DNA, and how they interact with other factors.

Spatial expression profiling using *in situ* hybridization is one approach to systematically examine gene expression patterns during development, and is a central assay in establishing regulatory links between TFs and regulatory elements. Large-scale embryonic mRNA expression pattern screens have been completed or are in progress for a number of model organisms including *C. elegans*, *C. intestinalis*, *D. melanogaster*, *G. gallus*, and *X. laevis*[7-14] and for specific tissue or developmental systems (for example, FlyTED, a *Drosophila* testis expression database, [15], and the Allen Brain Atlas of the adult mouse brain [16]). These large datasets provide a wealth of information about tissue and organ development, and can be used to find co-regulated genes and identify genes with similar functions [13,17,18]. Previously, as part of the ongoing Berkeley Drosophila Genome Project (BDGP) embryonic gene expression pattern genome-wide screen, we profiled 30% of the transcription factors with sequence-specific DNA-binding domains [13]. Reports in the literature (compiled by FlyBase [19]) include selected spatial expression data, either mRNA or protein, for 354 TFs. However, many TFs remain poorly characterized, and half lack any description of their embryonic spatial expression patterns.

A standard method to summarize spatial gene expression data is manual annotation using a controlled anatomical vocabulary (reviewed in [20]). For *Drosophila*, we developed a controlled vocabulary of 314 primary terms, described previously [13]. Anatomical terms that are part of a larger structure are linked by the ‘part of’ relationship and terms for structures that develop from one to another are linked by the ‘develops from’ relationship, so that the annotations track expression in developing tissues across stages. The primary terms can be generalized into a set of ‘collapsed terms’ that group rare or hard to distinguish terms with their more general parent term. The collapsed terms can be grouped further into organ systems to study organogenesis. The consistent annotation of expression patterns transforms the data into a format that enables integration with other genomic datasets and comparison of tissue expression patterns across species (4DXpress) [21].

We applied our large-scale *in situ* hybridization pipeline [13,22] to survey embryonic mRNA spatial expression patterns of all known and predicted *Drosophila* TFs (708) with sequence-specific DNA-binding domains (DBDs). This new dataset allows us to examine TF expression patterns in the context of 6,334 other genes profiled using the same experimental platform and annotation terms. We leveraged Model Organism ENCyclopedia Of DNA Elements (modENCODE) RNA-seq datasets to evaluate and validate properties of transcription factor mRNA expression. In contrast to previous studies, we examined the set of TFs expressed in the context of 16 organ systems, followed TF expression trajectories across development and identified regulators driving organogenesis. From our data we detected novel relationships and differences among transcriptional programs during organ system development. This is the first comprehensive TF study of this scale for any metazoan organism.

## Results

### TF predictions, expression patterns, and annotations

**Figure 1:**
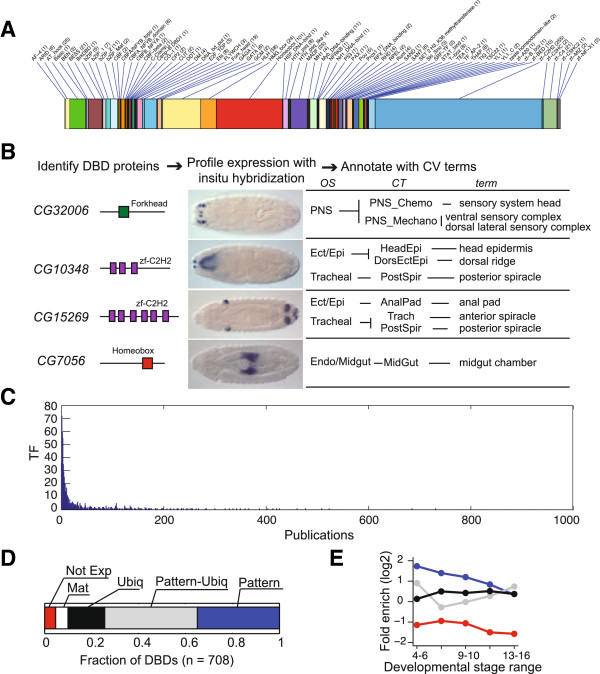
**Systematic profiling of TF mRNA embryonic expression patterns. (A)** Distribution of 708 TF genes by DBD class sorted alphabetically with the number in each class in parentheses. **(B)** Expression profiling pipeline and downstream analysis. Four examples of previously uncharacterized TFs showing the TF gene name, an illustration of the defining DBD, an *in situ* image for the gene, and annotations showing primary controlled vocabulary primary annotation terms (term), the associated collapsed terms (CT), and the organ system (OS). **(C)** Histogram showing the density of publications per TF as shown by the number of publications recorded in FlyBase TF gene reports (x-axis) in relation to the number of TFs (y-axis). Most TFs are concentrated around the x-axis origin, that is, referenced in a small number of publications. **(D)** Distribution of TFs into expression classes. We classified TFs as either not expressed (Not Exp), maternally deposited only (Mat: expression for gene observed at S1-3 and may persist for the next two stage ranges), ubiquitous-only (Ubiq: gene shows only ubiquitous staining at all stages expressed, with no anatomic terms annotated), patterned-only (Pattern: gene is assigned to a tissue term at all stages expressed), patterned-and-ubiquitous (Pattern-Ubiq: gene shows ubiquitous-only staining at one or more stages and pattern annotations in others, or a pattern with a weaker ubiquitous background). **(E)** Fold enrichment of fraction of TF *vs*. all genes (7,042) at each stage-range across expression classes. Color code for expression class is as in 1D.

In order to identify a complete set of DNA-binding transcription factors, we used InterProScan [23] to search for patterns of amino acids that matched a set of 71 putative DBD domain models collected from published TF annotation efforts [24,25] and from additional literature curation (Additional file 1: Table S1). Three TFs containing two additional DBDs not included in the protein domain databases were added manually (see Materials and methods). From this combination of computational predictions and literature curation, we identified 708 genes likely to encode sequence-specific DNA-binding TFs, representing a total of 73 DBD families (Figure 1 and Additional file 2: Table S2). The distribution of TFs by DBD domain is shown in Figure 1A. Of the 73 DBDs, 23 are present in more than five TFs. The zinc finger zf-C2H2 family is the largest group with 225 members, followed by the homeobox group (101 TFs) and the HLH group (58 TFs). Twenty DBDs are present in only one TF each.

To produce a comprehensive atlas of TF embryonic expression patterns, we designed experiments specifically to generate temporal profiles for the complete set of 708 TFs. For each TF we captured a standard set of whole embryo images at six morphologically distinguishable stage ranges. This complete set of images enhanced our ability to detect weak or highly restricted expression patterns and facilitated comparisons. We imaged and annotated embryonic expression patterns for 137 TFs not previously characterized in the literature, as well as 217 partially characterized TFs with no previous description of embryonic expression patterns.

The patterns were annotated with 314 controlled vocabulary terms [13]. We grouped these primary annotations into 127 parent collapsed terms, each of which is associated with an organ system (Figure 1B; Additional file 3: Table S3). Examples of expression patterns for all 16 organ systems are shown in Additional file 4: Figure S1. We validated expression when possible with primary literature. Although many *Drosophila* TFs had been studied previously, our survey of the literature revealed that 80% of the publications describe fewer than 20% of the TFs (Figure 1C). Even among the genes with published expression patterns, many were incompletely described. For example, we found previously unreported mesodermal blastoderm-specific expression of *regular (rgr)* (Additional file 4: Figure S1), and primordial photosensory expression of *Sno oncogene (Snoo).* The complete set of 708 TF expression pattern images and annotations is available at [26].

### TF expression profiles

We classified TFs using five expression pattern categories: not expressed, maternally deposited only, ubiquitous-only, patterned-only (defined with tissue-specific annotation terms at all expressed stages) or patterned-and-ubiquitous (defined with both tissue-specific and ubiquitous annotation terms). Nearly all TFs are expressed during embryogenesis (>96%), and most are expressed in a spatially restricted pattern sometime during embryogenesis (approximately 80%, Figure 1D). We examined these expression categories across all stages, and compared the distribution of the TFs to a set of 6,334 protein-coding genes, about half of the protein-coding genes in *Drosophila* (Additional file 5: Table S4).

The fraction of TFs expressed during embryonic development is very high and is significantly higher (*P* = 2.3 × 10^-46^, hypergeometric distribution) than the fraction of all genes. Only 3.5% (25 of 708) of the TFs are not expressed, compared to 22.5% of all genes, consistent with the concept of transcriptional regulatory cascades controlling development. This high proportion of TF embryonic expression (approximately 95%) has also been observed using abundance-based transcriptional profiling methods [27], and in spatial expression studies of TFs in the ascidian, *Ciona intestinalis*[9]*.* Although we detect maternal expression in 66.5% of TFs, only 4.8% of TFs are expressed solely as maternal transcripts (34 of 708) compared to 12.1% (*P* = 6.0 × 10^-10^) of all genes. Most of the maternally deposited TFs (92%) show zygotic expression, in contrast to 78% of all other genes.

Stage-specific expression classifications were used to determine whether more TFs are expressed than expected at each zygotic stage range. We found that the fraction of TFs expressed in a restricted pattern during early embryogenesis, 20.4% at stage range 4 to 6, is significantly (*P* = 3.1 × 10^-43^) greater than the 4.6% observed for all genes during this same period. This trend continues for all but the last stage range (Figure 1E; Additional file 5: Table S4).

The modENCODE embryonic RNA-seq time course [28] provides an independent measure of embryonic expression complementary to TF expression patterns captured by *in situ* hybridization. We used the RNA-seq scores to distinguish between failed experiments and negative spatial expression patterns (see Materials and methods). TFs not expressed in our *in situ* assay have the lowest embryonic RNA-seq expression scores, those with ubiquitous annotations show the highest expression scores, and genes with restricted patterns fall in between (see Additional file 6: Table S5). We have used these general trends on a case-by-case basis to evaluate our detected spatial expression patterns. However, a systematic quantitative comparison between the two datasets is confounded by two experimental differences, the strains and sampling parameters. RNA-seq samples were collected from a mutant strain (*y; cn bw sp*) in 2-h time intervals. The *in situ* data were produced using a wild-type strain, Canton-S that develops at a different rate. Our first four morphology stages represent small subsets within the early RNA-seq intervals, and our last two stages span multiple late RNA-seq intervals. We found that the spatial expression studies are qualitatively more sensitive than the embryonic RNA-seq studies for the developmental time course based on whole animal samples. Examples of TFs that show spatially restricted embryonic expression but very low RNA-seq scores include *PvuII-PstI homology 13 (Pph13)*, expressed in Bolwig’s organ, *Bteb2*, expressed in garland cells, and *RunxA*, expressed only in the visceral muscle of the posterior hindgut (Additional file 4: Figure S1).

For TFs that did not show embryonic expression by *in situ* hybridization, we analyzed RNA-seq expression in larval, pupal, and adult stages, as well as in dissected larval and adult tissue samples (Additional file 7: Figure S2). For 24 of these 25 TFs, little or no expression is detected in the embryonic RNA-seq time course samples corresponding to the periods studied by *in situ* hybridization. These TFs are primarily expressed in specific dissected postembryonic tissue samples including larval imaginal discs, larval, pupal, or adult CNS, or adult testes. Four TFs, *Visual system homeobox 2 ortholog* (*Vsx2*), *dissastisfaction* (*dsf*), *CG7786*, and *CG4374*, appear to be rarely expressed as they are not detected by these RNA-seq experiments.

### TF expression by organ system

**Figure 2:**
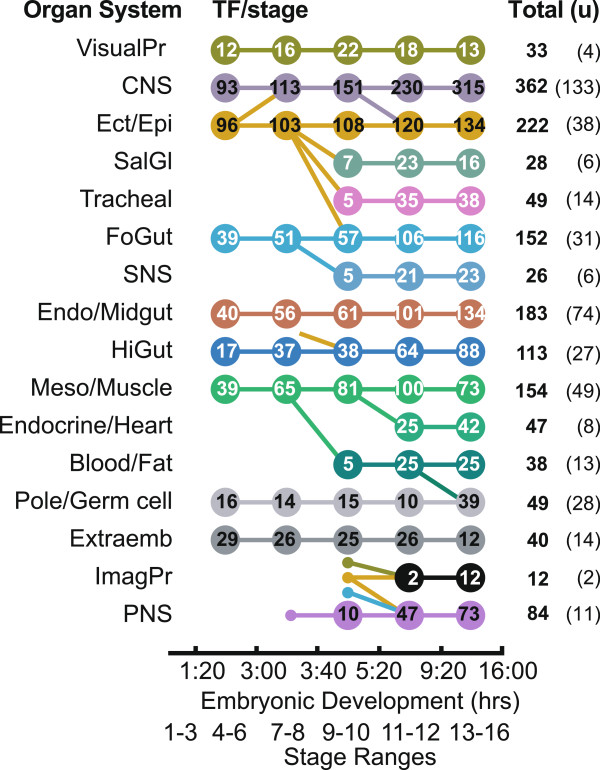
**Organ system relationships and TF count by organ system.** Number of TFs expressed at each stage for the sixteen organ systems. Colored circles show stage range when each organ system becomes detectable and connecting lines show developmental relationships between organ systems. Stage ranges are shown on the bottom line with corresponding developmental time in hours above. The two numbers on the right indicate the total number of TFs (Total) expressed in each organ system and the subset (u) representing TFs that were previously uncharacterized, or uncharacterized in embryonic development. See text for organ system descriptions.

To identify potential regulators of organogenesis, we focused our analysis on TF expression in the organ systems. Knowledge of the transcriptional programs that determine organogenesis is incomplete, as shown by our discovery that a significant number of previously uncharacterized TFs have spatially restricted expression patterns (206). The 16 organ systems, their developmental relationships, the number of expressed TFs by stage, the total number of TFs expressed in all stages, and the number of TFs with no previously described role in embryonic development are shown in Figure 2. The six major organ systems (central nervous system (CNS), ectoderm/epidermis (Ect/Epi), foregut (FoGut), endoderm and midgut (Endo/Midgut), hindgut (HiGut), and mesoderm/muscle (Meso/Muscle)) have anlage evident from the earliest zygotic stages. Specific anlage of the salivary gland (SalGl), tracheal system (Tracheal), stomatogastric nervous system (SNS), endocrine system and heart (Endocrine/Heart), blood and fat (Blood/Fat), imaginal primordia (ImagPr), and peripheral nervous system (PNS) develop from the major organ systems and are detectable starting in stages 9 to 11. Expression in the visual primordia organ system (VisualPr), which comprises the precursors of the adult optic lobes and the primordium of the adult eye, is detectable at all stages, as is expression in the pole cells and germ cells of the gonad (Pole/Germ Cell), and in the extraembryonic tissues (Extraemb), including the amnioserosa and yolk nuclei.

More TFs are expressed in the CNS than any other organ system, followed by Ect/Epi, Endo/Midgut, Meso/Muscle, and FoGut. The organ systems with the fewest expressed TFs are the SNS and SalGl, and the organ systems that form adult primordia, the ImagPr and VisualPr. Among the TFs expressed in neural organ systems, most of those expressed in the VisualPr, PNS, and SNS are well characterized, whereas more than one-third of the TFs expressed in the CNS had not been studied previously (133 TFs). Similarly large numbers of TFs expressed in the Endo/Midgut (40%) and Meso/Muscle (32%) were not previously described, as well as more than half of the TFs expressed in Pole/Germ cell organ system (57%).

For most organ systems the number of TFs expressed increases during development, consistent with a model of hierachical TF activation. Exceptions are: the VisualPr, Meso/Muscle, and SalGl organ systems, where the number of expressed TFs peaks at stages 9 to 10 or 11 to 12; the PoleGerm cell organ system, where the number of TFs declines until stages 13 to 16 but then spikes as expression in the gonads becomes detectable; and the Extraemb organ system, which does not form larval organs and where the number of TFs expressed decreases by half by stages 13 to 16.

**Figure 3:**
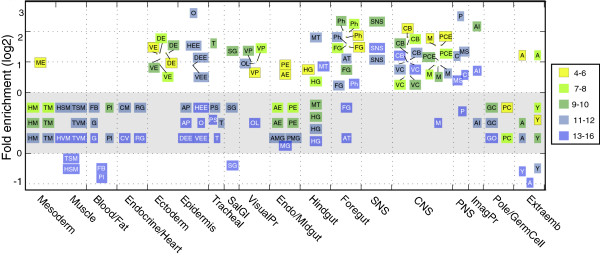
**Summary of TF enrichment within collapsed tissue annotation terms.** Enrichment of the fraction of TFs *vs.* All genes for collapsed annotation terms at each stage-range. Each annotation term is represented as a box, where the y-axis value is the fold enrichment (FE) and the color indicates the stage range. To highlight the organ system trends, collapsed tissue annotations are grouped by organ systems shown on the x-axis. All the annotation terms that did not show significant enrichment (FDR >0.05) are assigned a fold enrichment of zero and placed in the grey area of the plot. For the Ectoderm, VisualPr, CNS, and Foregut organ systems, the positions of the annotation term boxes are adjusted and linked to the actual FE value, represented as a point. Individual collapsed tissue annotation abbreviations and annotation term mappings are detailed in Additional file 3: Table S3. Short key to terms: ME: mesoderm; HM: head mesoderm; TM: trunk mesoderm; HSM: head somatic mesoderm; HVM: head visceral mesoderm: TSM: trunk somatic mesoderm; TVM: trunk visceral mesoderm; FB: fat body; Pl: plasmatocytes; G: garland cells; CM: cardiac mesoderm: CV: cardiovascular system: RG: ring gland; DE: dorsal ectoderm; VE: ventral ectoderm; HEE: head epidermis; DEE: dorsal ectoderm/epidermis; VEE: ventral ectoderm/epidermis; AP: anal pad; O: oenocyte: T: trachea; PS: posterior spiracle; SG: salivary gland; VP: visual primordium; OL: optic lobe; AE: anterior endoderm; PE: posterior endoderm; AMG; anterior midgut: PMG: posterior midgut; MG: midgut; MT: Malpighian tubules; H: hindgut; Ph: pharynx; FG: foregut; AT: atrium; SNS: stomatogastric nervous system; PCE: procephalic ectoderm; CB: central brain: VC: ventral nerve cord; M: midline; P: PNS-photosensory: MS: PNS-mechanosensory; C: PNS-chemosensory; AI: adult imaginal primordium; PC: pole cell; GC: germ cell; GO: gonad; A: amnioserosa: Y: yolk.

We showed that TFs with tissue-specific patterns are over-represented (enriched) in early stages of development (Figure 1E). To expand on this observation, we used the collapsed term annotations to determine whether any of the TFs are enriched or under-represented (depleted) relative to the total set of characterized spatially expressed genes for each organ system. We find that TFs are enriched at the beginning of most lineages, with the notable exceptions of Meso/Muscle, Blood/Fat, Endocrine/Heart, and Pole/Germ cell (Figure 3). In the CNS, TFs are enriched at all embryonic stages. The CNS enrichment findings are consistent with previous reports based on data from approximately half of the TFs [13,27]. This observation holds when all TFs are considered. In particular, we observe enrichment of TF expression in the two major components of the CNS, the central brain and ventral nerve cord, but not in the late embryonic midline, where axon connections that are formed rely on cell-cell signaling. Interestingly, TFs are depleted in the late developmental stages of embryonic muscle, fat body, plasmatocytes, salivary gland, and the extraembryonic tissues of the yolk and amnioserosa.

### TF expression pattern associations with DNA-binding domain classes

**Figure 4:**
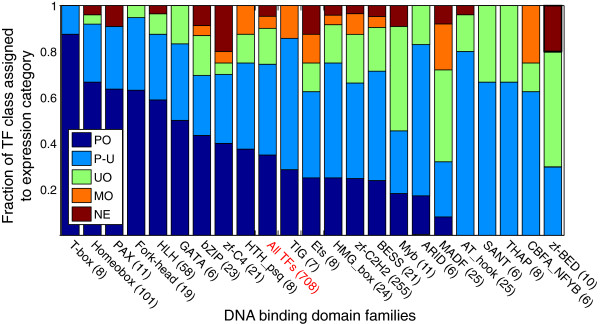
**Expression properties of DBD families.** Expression categories (PO: patterned only, P-U: patterned-and-ubiquitous, UO: ubiquitous only, MO: maternally deposited only, NE: not-expressed; see Figure 1 legend for descriptions) for 22 DBD families with more than five members each, ordered by fraction of TFs classified as PO. For the purposes of this analysis, bZIP, bZIP_1, and bZIP_2 are grouped as a single DBD family. The number of TFs in each DBD family is indicated in parentheses after the DBD name. For reference, an additional column (All TFs) shows the distribution of all 708 TFs by expression category. See Additional file 1: Table S1 for more information about DBD names, with Pfam and InterPro identifiers.

We characterized expression patterns of TFs as a function of their DBDs. Of the 73 DBDs, 23 have more than five members. For this set, we determined the distribution of each DBD using the expression pattern categories and found that all TFs containing a T-Box, and the majority of TFs (>75%) containing Homeobox, PAX, Forkhead, HLH, GATA, TIG, and AT_hook DBDs show spatially restricted expression consistent with their prominent role in organ system specification (Figure 4). A large fraction of the TFs containing MADF and zf-BED are ubiquitously expressed. We also assessed the association of protein domains with specific organ systems against the null of independence. At a false discovery rate of 10%, no domains were significantly associated with any organ systems. Even though it has been suspected, our complete dataset establishes that individual DBD families are not used for specific organ system differentiation.

### Insights into organ system development and differentiation

**Figure 5:**
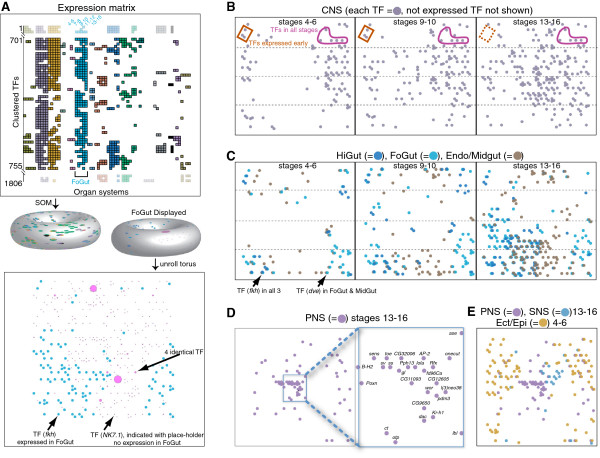
**Self-organizing maps (SOM) of TF expression in organ systems. (A)** Portion of the TF expression matrix showing expression (colored boxes) in the appropriate stage ranges for each organ system and highlighting a section (54 of 152 TFs) of the foregut (FoGut) organ system cluster (blue). Rows are TFs and columns are organ systems, with a separate column for each stage range within an organ system (colors identical to Figure 2). The entire expression matrix is used as input to generate the SOM toroidal map. The toroid on the left displays the complete expression composite of all TFs expressed at stage range 13 to 16 and the toroid on the right highlights the FoGut TFs (blue) at the same stage range. The distribution of the FoGut TFs (blue filled circles) in two dimensions is shown in the lower panel. Co-expressed TFs (across all organ systems and all stages) will end up close to each other. **(B)** CNS TF map showing the dynamics of TF expression in a single organ system during development. The overall number of filled circles (=TFs) increases significantly at later stages, highlighting increased number of TFs and TF complexity. Two examples for TFs with different temporal dynamics are highlighted. **(C)** TF expression in the Hindgut, Foregut, and Endoderm/Midgut organ systems highlighting the relationships between the three organ systems. Intersecting filled circles (arrows show two examples) indicate expression in multiple systems; for example, at stages 4 to 6 only six TFs are co-expressed Hindgut and Foregut, while co-expression in midgut and hindgut/foregut is more common. **(D)** TFs in the PNS at stages 13 to 16 (center panel). The boxed region is expanded to the right. **(E)** Distinct and overlapping groups of TFs active in the PNS and SNS at stages 13 to 16 superimposed with the TFs active in the ectoderm/epidermis stages 4 to 6.

We assessed TF expression in each organ system at each stage range to create an expression matrix for the 535 TFs with patterned zygotic gene expression (Additional file 8: Table S6). The TFs are clustered by their expression in specific organ systems. For each TF the matrix also displays all concurrent organ system expression. The expression profiles for the 16 organ systems are summarized in text descriptions, including identification of TFs with newly characterized expression, in Additional file 9. For a systems overview of TF relationships, we used the organ system matrix to identify co-expressed TFs during organ system development and visualized the groupings in a summary map. To generate the map we used the Self-organizing map (SOM) algorithm with a novel visualization strategy (Figure 5).

The SOM algorithm reduces the complexity of the high dimensional organ system expression matrix by placing the TFs onto a two-dimensional (2D) surface with a clustering procedure similar to k-means, while adjusting the surface to the shape of the TF data cloud (reviewed in [29]). The result is a 2D representation of the TFs in a toroid structure that arranges TFs with similar organ system profiles close to each other and dissimilar TFs further apart. For display, the toroid is opened and flattened, so that positions at opposite edges of the map correspond to positions adjacent on the toroid (Figure 5A). Like co-expression analysis [30], SOMs identify correlated expression patterns while adding a statistically defined and optimized grouping of the correlated TFs into connected nodes that allow for visualization in 2D. For clarity, connections between the nodes are not shown.

In a novel use of SOMs, we created dynamic overlays by placing a color-coded label on a TF if it is expressed in a given organ system at a given stage. All SOM displays are based on the entire expression matrix and have identical TF data point locations, but differ in the organ system overlays (Figure 5). In addition, we provide an interactive SOM viewer [31], allowing visualization and exploration of the TFs expressed in any combination of organ systems and stages.

We found that most, but not all, organ systems are characterized by the expression of distinct groups of TFs with similar expression patterns, as illustrated by their positions in distinct regions in the SOM (examples in Figure 5A). Organ systems with well-defined map distributions of TFs are the early activated organ systems: the neural (CNS, SNS, and last two stages of PNS), gut (FoGut, Endo/Midgut, and HiGut), Ectoderm/Epidermis, Mesoderm/Muscle, and Pole/Germ cells. In contrast, TFs expressed in the salivary glands, imaginal primordia, and endocrine system or heart show little overall expression pattern similarity, as illustrated by their widely dispersed positions in the SOM (Additional file 10: Figure S3).

We examined TF expression dynamics within each organ system across developmental time, and found three classes of TF expression: (1) TFs expressed only in early development; (2) core sets of TFs that persist across stages; and (3) larger sets of TFs expressed late in development. All three classes are illustrated graphically for the CNS at three stage ranges (Figure 5B).

We examined functionally related organ systems to identify the complete set of TFs governing the development each organ system and compared the sets of TFs expressed in different organ systems. We created an overlay for the SOM indicating whether a TF had been previously characterized, allowing us relate the TFs with newly described expression patterns to previously described TFs with similar expression patterns. Here we focus on two sets of related organ system groups, the three gut organ systems, and four neural organ systems.

We compared the dynamics of TFs expressed in the functionally related organ systems foregut, hindgut and midgut (Figure 5C). TFs expressed in these three gut organ systems are distinct from those expressed in the CNS, as shown by their positions on the map in regions well separated from the positions of TFs expressed in the CNS. All three gut organ systems show expression of exclusively early TFs, persistent TFs, and increasingly diverse sets of TFs over time, similar to the TF classes observed for the CNS. Despite their common ectodermal origin, in early development, foregut and hindgut express only six TFs in common, whereas the endoderm derived midgut shares expression of more than three times as many TFs with either foregut or hindgut. Only in late development, stages 13 to 16, do we observe more substantial overlaps. The hindgut appears to be the least distinct of the three gut organ systems at this stage, with two- to four-fold more hindgut TFs co-expressed in one of the other gut tissues than TFs expressed in either the foregut or midgut. However, one well characterized TF, *brachyenteron (byn)*[32], is expressed exclusively in the hindgut throughout embryonic development. No such specific TF is expressed in either the foregut or midgut.

Among the four neural organ systems, CNS, PNS, SNS, and Visual Pr, we detected groups of TFs expressed uniquely in the CNS, PNS, and SNS. In the SNS only seven TFs are not co-expressed in the CNS or PNS, and one (*doublesex-Mab related 93B* (*dmrt93B*)) is expressed only in the SNS. Of the 84 TFs expressed in the PNS, all but nine TFs are expressed concurrently in the CNS, and five TFs (*spineless* (*ss*), *cousin of atonal* (*cato*), *Pph13*, *Sox box protein 15* (*Sox15*), and *CG32006*)) are specifically restricted to the PNS. Most of the seven previously undescribed TFs in the PNS appear panneural, that is, most likely involved in differentiation, with the exception of *CG16815*, which appears early during both PNS and CNS development. The uncharacterized TF *CG32006* (Figure 1B and 5D*)* is closely associated on the map with a small group of well-known PNS regulators, including *ss*, and a broader group of TFs expressed in the PNS includes four other TFs, *CG11093*, *CG12605*, *CG9650*, and *pou domain motif 3* (*pdm3*), previously uncharacterized in embryogenesis (Figure 5D)*.* The CNS expresses the largest number of TFs (362 TFs) and is the most distinct of the three nervous systems, with 81 TFs not expressed in any other organ systems in late development. The expression patterns for more than half (45) of these CNS specific TFs were uncharacterized previously.

In contrast, no single TF is expressed exclusively in the adult VisualPr. Nearly all TFs expressed in the VisualPr also are expressed in the Ectoderm/Epidermis, or the CNS, or both, consistent with the ectodermal origin of these tissues. The one exception is the well-characterized TF *glass* (*gl*) [33], which is required for normal photoreceptor development during metamorphosis [34]; in the embryo it is expressed in the adult optic lobe anlage (VisualPr), in Bolwig’s organ (larval eye, considered part of the PNS, Collapsed Term: PNS_Photo), and in the corpus cardiacum (Endocrine/Heart). The smaller number of TFs expressed in the VisualPr (33) allowed us to closely investigate a temporally sorted graph (Additional file 11: Figure S4). One-third of the visual system TFs are activated early, one (*charlatan* (*chn*)) persists across all stages, and 12 are expressed only in the last two stages, consistent with our earlier observation that organ system development is characterized by expression of a core set of TFs expressed early, with dynamic additions and subtractions of TFs during other stages.

We identified TFs that are expressed early in one organ system and later in other organ systems. TFs expressed in the early ectoderm are re-deployed later in the anlage of the PNS and SNS (Figure 5E). Specific TF expression in the CNS precedes the expression of the same TFs in the PNS, whereas TFs expressed in both CNS and SNS occur in parallel. TFs expressed late in the PNS are distinct from those expressed in the CNS (data not shown) or SNS (Figure 5E).

**Figure 6:**
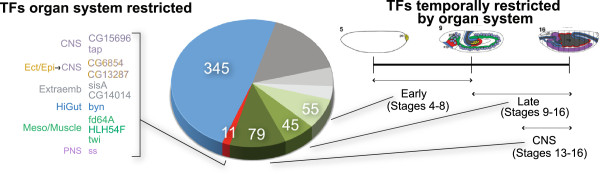
**Distribution of TFs as a function of their expression profiles.** The colored regions of the pie chart highlight the TFs categorized as consistently expressed in a single organ system (red), temporally restricted early or late in embryonic development to a single organ system (shades of green), or expressed in multiple organ systems (blue). The segments of the pie chart shaded in grey from light to dark are: not expressed (25 TFs), maternally expressed only (34 TFs) and ubiquitously expressed only (114 TFs), respectively. TFs restricted to a single organ system both early and late are listed on the left with the associated organ system. The time line on the right shows the division of early and late development with diagram illustrating development of organ systems at stages 5 (early body plan determination and organ specification), 9 (organ specification nearly complete), and 16 (organ system differentiation).

Our interrogation of TF expression in all organ systems across developmental time showed that over half (345 TFs) of all TFs with patterned expression are expressed in multiple organ systems at any given stage, consistent with models of TFs acting cooperatively in dynamic groups. In addition, our results indicate that many TFs (113) are expressed in only a single organ system including eleven TFs that are expressed in every stage throughout embryonic development (Figure 6). Seven of these have been previously described and implicated in cell fate maintenance, suggesting a similarly critical function for the four previously uncharacterized TFs in this set. By dividing development into early (stages 4 to 8) and late (stages 9 to 16), that is, into predominantly organ system specification and organ system differentiation, we find that the 179 patterned TFs are restricted temporally to a single organ system (Figure 6). For example, we identified a set of four TFs (*senseless-2* (*sens-2*), *Ecdysone-induced protein 75B* (*Eip75B*), *Estrogen-related receptor* (*ERR*), and *CG7056*) expressed only in the midgut (and more specifically the midgut chamber) at stages 11 to 16, including one previously uncharacterized TF *CG7056* (Figure 1B). We also identified a set of seven TFs (*RunxA*, *Chorion factor2* (*Cf2*), *CG6689*, *CG17181*, *CG13424*, *Meiotic central spindle* (*Meics*), and *nautilus* (*nau*)) restricted to the Meso/Muscle organ system at stages 11 to 16. Of the 55 TFs restricted to a single organ system early, all but two are expressed in multiple organ systems late, while 38 of the 45 late restricted TFs (excluding CNS TFs) never show expression in another organ system. By far the largest group of TFs restricted temporally to a single organ system is the set of 79 TFs expressed exclusively in the CNS late in development, indicating a highly specific role of these TFs in CNS diversification. This group includes 62 TFs that show no expression until late in development when they appear only in the CNS, and another 17 TFs that show expression in multiple systems early but resolve exclusively to the CNS late. These data provide evidence that organ system development requires a set of TFs that are expressed in various organ systems in distinct and dynamic combinations, as well as TFs with expression temporally restricted to a single organ system.

## Discussion

### Assembly of a TF reference expression pattern dataset

We determined embryonic TF spatial expression patterns in order to generate a comprehensive dataset and to identify developmental TF networks for each organ system in the body. Specifically, we focused our expression studies on the core set of regulators formed by TFs representing all known sequence-specific DNA-binding motifs, because these TFs confer tissue specificity and drive enhancer activity. Substantial community effort has gone into defining sets of TFs based on DBD motif models and experimental evidence [24,25,35-37]. The TF list compiled for this work includes all 212 TFs found in the FlyFactorSurvey list [36], and incorporates nearly all of the highest scoring FlyTF candidates [35]. While our list shows strong correspondence to other *Drosophila* TF lists, it has been curated to represent the most comprehensive set of TFs at the point of this study. Our computational search identified additional factors for families with well characterized DBDs, and our manual curation identified TFs with DBDs described in the literature but not included in the Pfam DBD models, such as the C-clamp [38] and the zeste homeodomain-like DBD [39]. We excluded proteins likely to act as chromatin remodelers or that have only protein-interacting domains, as well as general transcription factors that bind to common proximal promoter elements and interact with the basal transcription machinery. Although we recognize that these classes of proteins have important roles in basal transcription or repression of transcription, they are not the primary drivers of regulated, tissue- and organ-specific transcription initiation. Certain domains, for example, the homeodomain, clearly define a protein as a sequence-specific DNA-binding TF; however, this determination is less straightforward with other DBDs (especially the zinc finger C2H2 domain), which are known to have other functions, including binding RNAs and proteins [40]. The precise role of many TF candidates remains unclear, and determination of their spatial expression patterns is a crucial step towards an understanding of the development and differentiation of the embryo.

Our dataset of TF expression pattern images and annotations is searchable by annotation term and by image similarity. The TF expression data of this specific effort are now incorporated in our larger database of expression patterns, currently representing over 7,600 genes, and data collection continues for additional genes, including other types of regulatory genes and potential TF targets. As an addition to the expression and annotation resource we also provide an interactive self-organizing map, which enables exploration by organ system, stage, and degree of characterization [31].

### Classification of TFs

Compared to current computer- and image-based annotations, our manual annotations provide a more accurate description of the terminally differentiated stages of embryogenesis, producing a more reliable source for generating insights into the dynamics of organ development. By means of gene enrichment analysis we identified temporal and spatial waves of concerted TF activity. We used our annotations to divide the dataset into tissue-specific (patterned) and ubiquitously expressed TFs. Although we focus here on TFs with patterned expression, ubiquitous TFs also have critical developmental roles. For example, *daughterless* (*da*) is a ubiquitously expressed TF that dimerizes with other HLH TFs and activates expression of neuron-specific genes [41], and with the HLH TF *twist* (*twi*) forms a heterodimer that can switch from an activator to a repressor over the course of mesoderm and muscle development [42]. Patterned TFs are significantly enriched early in development (stage range 4 to 6) when tissue specification programs are initiated. These are most likely to be the TFs that determine the basic body plan and initiate the formation of each organ system. Although classic genetic screens [43,44] identified many TFs required for body plan specification at developmental stages 4 to 6, we identified 35 TFs with previously unreported patterned expression in early zygotic development. Efforts to refine models of blastoderm gene regulatory networks will benefit from inclusion of not only these additional TFs but from the full complement of 192 TFs with patterned expression in the early zygote.

### Insights into organ system regulatory networks

Spatial expression data for the complete set of TFs allowed us to track expression trajectories across development, compare organ systems, and identify potential new members of regulatory networks. Our investigation into the development of the organ systems revealed that the major organ systems are characterized by expression of distinct combinations of TFs. We found core sets of TFs that are activated early in organ system lineages and remain on throughout organ system development, as well as other sets of TFs that are required to initiate organ system specification and then are turned off, as illustrated for the visual system. Although TFs are enriched in early development, the absolute number of TFs expressed increases later in the development in most organ systems (11 of the 16), consistent with organ system diversification and increases in the numbers of non-TF genes expressed as well as TFs. The enrichment of TFs relative to other genes is maintained throughout development only in the neural organ systems, such as CNS and PNS.

We compared the TF expression dynamics of three neural organ systems and the three organ systems that form the gut. The nervous systems (CNS, SNS, PNS) are derived from closely related cell lineages with similar TF expression profiles, but the three organ systems diverge late in development, consistent with their functional diversification. The CNS in particular expresses a large number of uniquely expressed TFs (79) by terminal differentiation. In contrast, the three gut organ systems (foregut, hindgut, and midgut) arise from divergent origins and express discrete TF sets early. As development progresses the expressed TF sets become more similar as the unified gut coalesces. The two ectodermally derived gut systems, the foregut and hindgut, express fewer TFs in common with each other than each shares with the midgut, indicating that even closely related tissues do not necessarily share the same transcriptional programs.

We found the Pole/Germ cell organ system to be particularly unusual. TFs are not enriched in this organ system at any stage, and TF diversity does not increase steadily over embryonic development as seen in most other organ systems. With over half of the Pole/Germ cell TFs previously uncharacterized, this organ system may prove a particularly interesting area for further investigation.

Studies of TF regulation of the development of particular tissues (for example, cardiac cell fate [45]) have shown that TFs act collectively to activate enhancers, and previous co-expression analysis, based on the collapsed terms for approximately half of the TFs from our current dataset, indicated significant plasticity of co-expressed TFs, with nearly all possible TF pairs occurring together in at least one tissue and stage [27]. Our observation that most patterned TFs show complex expression in multiple organ systems is consistent with the idea of dynamic combinatorial TF function. Ultimately, demonstration of co-expression at the level of cooperative binding to regulatory elements will require higher resolution imaging or biochemical studies. An unexpected finding is the large number of TFs that show organ system specificity, particularly considering that our probes for these experiments are derived from full-length cDNA clones and in most cases will detect all transcript isoforms. The organ system specificity of individual TF transcript isoforms resulting from alternative promoters, alternative splicing or alternative 3′ ends, may be even greater than that which we detect using these common probes. We found that a small number of TFs (11), including well known cell-type and tissue-specific regulators of transcription such as *byn*, *ss*, and *twist*, and four uncharacterized TFs, are restricted to a single organ system across all stages of embryogenesis. However, if early and late embryonic developmental stages are considered separately, the number of TFs with expression specific to a single organ system expands to 190 TFs. Primarily but not exclusively in the CNS, we find clusters of TFs that show common organ system specific expression across two or more stage ranges. In particular, many of the CNS specific TFs have not been previously characterized. Most of the studies to date have focused on the TFs that control development of the CNS, whereas fewer have focused on the differentiation of the CNS. In general, more of the uncharacterized TFs are expressed later in development, emphasizing that our understanding of early patterning is more robust than our understanding of the later stages of embryonic development.

## Conclusions

Expression patterns for sequence-specific TFs comprise a critical biological dataset because of the central role of TFs in transcriptional regulatory networks that control the development of multicellular organisms. We used DNA-binding protein domains to identify a comprehensive list of 708 sequence specific TFs in *Drosophila* and performed experiments specifically to generate a complete reference data set of spatial expression patterns during embryonic development. Despite the importance of TFs, most publications focus on a relatively small number of TFs, and even previous large surveys included only half of the TFs in this dataset. Spatial expression patterns for 354 TFs are described for the first time in our study. Even in the most intensively studied embryonic stage (that is, blastoderm) and well-characterized organ systems (for example, Meso/Muscle or CNS), we identified new regulatory players.

Consistent with the view of pervasive combinatorial TF action, we find that most TFs with patterned expression are expressed dynamically in multiple organ systems (79%). The remaining 21% of the patterned TFs are expressed in only a single organ system during either organ system specification (early) or differentiation (late). We conclude that TF regulatory networks utilize both promiscuously expressed TFs and uniquely expressed TFs with roles devoted to the development of individual organs.

From our examination of TF expression in organogenesis, we discovered new associations of TFs co-expressed in organ-specific regulatory networks, suggesting candidates for further investigation through genetic and biochemical studies and higher-resolution imaging [46]. The images from our collection are a new resource for image-based analysis, which can be used to identify expression pattern components and relationships not captured in the annotations [18], and extended to include the analysis of *cis*-regulatory modules (CRMs) proximal to TFs [47]. Our complete dataset and our focus on TF expression in the major organ systems allow new TF network analysis and investigation into the role of TF combinatorial complexity in organogenesis.

## Materials and methods

### Prediction of DBD genes

We implemented InterProScan [23] to search for proteins with matches to a list of 71 Hidden Markov Models that correspond to DBDs (Additional file 1: Table S1). When available, we used the Pfam models [48] which for zf-C2H2 DBDs are more restrictive than the models used by other protein domain databases such as SMART [49] or Prosite [50]. We used alternative models for the DBDs that do not have Pfam models and for the MADF domain, which was not present in the InterProScan v4.4 download. BEN [51], Bzip [52], zeste homodomain-like [39], and C-clamp [38,53,54] domain family TFs were curated from the literature. This combination of computational pipeline and manual curation identified 708 putative DBD genes (Additional file 2: Table S2). Zinc finger domains zf-CCCH and zf-DHHC were not included in our list of DBD models, as they are associated with protein-protein interactions and palmitoyltransferase activity, respectively.

### *In situ* hybridization and image capture

Methods for *in situ* hybridization and image capture have been described previously [8,13,22]. For the TF set, wherever possible we captured a minimal set of seven images for every experiment (one stage 0 to 3 and dorsal and lateral views of stages 4 to 6, 9 to 10, and 13 to 16), with additional images as needed to capture dynamic or complex patterns. Expression patterns were validated by comparison to RNA-seq and any available primary literature. The *in situ* experiments were repeated in cases of conflicting data. Images were captured using a Zeiss Axioplan microscope equipped with a SPOT RK1 camera, or a Zeiss Axioimager equipped with a SPOT Flex camera. The images shown in the figures were downloaded from our database, with minor color balance changes to correct for variations in camera performance. To facilitate computational image analysis, each embryo image was converted into a Triangulated Image (TI), which is a standardized digital representation of the expression pattern [18], also available for download from [26]. Probes were generated as follows: for 684 genes, we used the BDGP cDNA clone collection as the source of templates; for five (*CG7045*, *CG33557*, *slou*, *pb*, *Dbx*), we used clones provided by Bart Deplancke and Korneel Hens at Ecole Polytechnique Federale de Lausanne, Switzerland [55]; and for genes not represented by a clone (19 TFs), templates were generated by single exon PCR amplified from genomic DNA with a T7 RNA polymerase tailed reverse primer.

### Pattern annotation

The strategy for assigning the primary annotations from the controlled vocabulary (CV) and subsequent grouping of the CV terms into collapsed terms and organ systems has been described [13]. Organization of CV terms by collapsed terms and organ-systems used in this study is shown in Additional file 3: Table S3. The complete set of annotations for all genes used in this study is available from [26].

#### Statistical analysis of enrichments

In order to test statistical associations between categorical classifications we built two by two contingency tables and implemented Fisher’s exact test to calculate a *P* value. For a contingency table with four values (a, b, c, and d) the probability follows the hypergeometric distribution, where p = ((a + b)! (a + c)! (c + d)! (b + d)!)/(a! b! c! d!). *P* values were corrected for multiple hypothesis using the Benjamini–Hochberg procedure for false discovery rate control. For visualization, we calculated a fold enrichment statistic, where the observed proportion of a classification within the transcription factor class was scaled by the expected proportion, determined from all genes.

### Transcriptional profiling data analysis

For comparison of the mRNA *in situ* pattern annotations and other transcriptional profiling platforms, we utilized two large-scale studies, including an RNA-seq developmental time-course with 30 sample points [28], and 29 dissected tissues from larval, pupal, and adult stages [56]. The *in situ* hybridization data stage ranges do not correspond well with the RNA-seq developmental timepoints; therefore we used the following scheme to roughly correlate time points to the morphological stage ranges. RNA-seq sample em0-2 hr maps to *in situ* stages 1 to 3, em2-4 hr to *in situ* stages 4 to 8, em4-6 hr to *in situ* stages 9 to 10, em6-8 hr and em8-10 hr both to *in situ* stages 11 to 12, and em10-12 hr, em12-14 hr, em14-16 hr, and em16-18 hr all map to *in situ* stages 13 to 16. Formation of the cuticle around 16 h after egg laying makes expression unlikely to be detectable by *in situ* hybridization after stage 16. Accession numbers and sample descriptions for the RNA-seq data are provided in Additional file 12: Table S7.

### Data visualization by a self-organizing map

To generate the organ system expression matrix we used the previously defined mapping of collapsed terms to organ system for each of the six stage ranges. We created a matrix with rows for each TF that is expressed in an organ system at least one of the five zygotic stage ranges, and color-coded columns for the organ systems and stages. For each of the 16 organ systems we filtered and grouped the entries. To improve visualization, we used hierarchical clustering with hamming distance and complete linkage to group similar TFs to each other. Concatenating all 16 organ systems into one combined matrix generated the final TF expression matrix (Additional file 8: Table S6).

We used the unique rows (TFs) of this matrix and generated the SOM using the Matlab somtoolbox [57]. The SOM method seeks a labeling function f that assigns a cluster to each observation while forcing the cluster centroids with respect to a topographic structure M. Based on empirical tests, we selected a 20 × 20 toroid grid for M. The SOM algorithm minimizes following objective function L:

$$L\left(f,m\right)=\sum_{J=1}^{N}\sum_{k\in M}h\left(f\left(j\right),k\right){||x_{j}-m_{k}||}_{2}^{2}$$

*f(j)* is the distance between the data point *x*_*j*_ and the cluster centroid *m*_*k*_, *h* is a neighborhood decaying function of M. For *h* we used following Gaussian-like decay function:

$$h_{t}\left(i,k\right)=\exp\left(-\frac{d_{M}{\left(i,k\right)}^{2}}{2\sigma_{t}^{2}}\right)$$

*d*_*M*_*(i,k)* was set to the Euclidean distance between *i* and *k* on *M*. Our distance metric on the data space was set to one-minus-correlation distance. To initialize the cluster centroids, we performed PCA on the TF expression matrix and set the centroids to the grid on a 2D plane of the first two major components. We linearly decayed the neighborhood size σ_t_ from 5.0 to 0.5 during the first 400 iterations and left σ_t_ constant at 0.5 for the final 100 iterations.

We validated the resulting SOM layout by generating a similar map with a Minimum Spanning Tree algorithm and a map with a force based graph layout as proposed by Fruchterman and Reingold (data not shown) and found the three maps similar with the SOM map being the most compact and informative. We also validated our SOM layout with the newer t-SNE visualization and found no significant changes in the overall layout [58]. As additional validation, we performed a stability analysis by removing a random set of TFs in each tissue. No significant changes were detected in the overall map structure, indicating a stability of our results for potentially missing data.

To visualize the resulting SOM, we uploaded the data into a custom web-viewer (Frise, unpublished) and saved selected overlay combinations for Figure 5. Overlays were generated either from the TF matrix or a manually curated list of characterized TFs.

## Abbreviations

BDGP: Berkeley drosophila genome project; CNS: Central nervous system; CRM: *cis*-regulatory module; DBD: DNA binding domain; PNS: Peripheral nervous system; SNS: Stomatogastric nervous system; SOM: Self-organizing map; TF: Transcription factor.

## Competing interests

The authors declare that they have no competing interests.

## Authors’ contributions

SEC designed and managed the project. SEC, CAB, and ASH curated the TF list. WWF, RW, and ASH prepared embryos and imaged and annotated expression patterns. RW prepared probes and conducted all embryonic *in situ* hybridizations. ASH managed production. VH participated in the mapping of controlled vocabulary to organ systems, annotated expression patterns, and reviewed the annotations. CAB, EF, SW, MK, and BY developed statistical analysis methods; SW and EF produced the SOM visualizations and interactive map. CAB, SW, EF, ASH, and SEC analyzed the data. EF administered computer infrastructure for data storage, distribution, and analysis. ASH, CAB, EF, and SEC wrote the manuscript with input from all authors. All authors read and approved the final manuscript.

## Supplementary Material

Additional file 1: Table S1 InterPro, Pfam, and hand curated DNA-binding domains used to generate the TF list used in these studies. Click here for file

Additional file 2: Table S2 List of sequence-specific *Drosophila* TFs and their associated DNA binding domains. Columns A-D show the FlyBase identifiers, gene symbols, and synonyms for the 708 TF genes described in this study and their associated DNA binding domains (DBD). Shown in columns E and F are the corresponding FlyTF final calls and scores, or NA for genes not annotated as a TF in the FlyTF list of 754 putative TFs [59]. Click here for file

Additional file 3: Table S3 Correlation of controlled vocabulary terms, collapsed terms, and organ systems. Mapping of the names and abbreviations for CV (controlled vocabulary), CT (collapsed terms), and OS (organ system) and the stage ranges to which the terms apply are shown in adjacent columns. Abbreviations for CT terms (column D) are used in Figure 3. Terms that have no true organ system association (for example, ‘maternal’ and ‘ubiquitous’) are indicated by #N/A, but ubiquitous is arbitrarily assigned an organ system number of 17. Click here for file

Additional file 4: Figure S1 Examples of TF expression patterns representing the 16 organ systems. Click here for file

Additional file 5: Table S4 Expression category by stage range for all genes in this study. The ‘Gene set’ entry (column C) indicates whether the gene is from the list of 708 TFs (TF) or is from the remaining genes in the Release 3 BDGP expression pattern collection (non-TF). Expression classes are as described in Figure 1. The entry under ‘All stages’ (column D) is the summary classification for each TF and is used in the counts shown in Figure 1D. The separate classifications for each of the six stage ranges were used to determine the enrichment by stage shown in Figure 1E. Click here for file

Additional file 6: Table S5 RNA-seq scores for the embyronic time course samples for each TF with stage-by-stage *in situ* expression pattern categories. Click here for file

Additional file 7: Figure S2 Gene expression profiling of 25 TFs not detected spatially in embryos. Click here for file

Additional file 8: Table S6 Expression matrix clustered by organ system. Following the TF gene name, FlyBase identifier, and DBD, columns record expression of each TF in each organ system by stage range. The organ system color code is shown in Figure 2 and the number of columns for each organ system reflects the number of stages where the organ system is morphologically detectable (Figure 2). Columns are ordered by organ system according to the organ system number code shown in Table S3 (Additional file 3). A colored box indicates expression and clicking on a colored box will show the number code for the organ system; clicking on a white box will show 0 for not expressed in that organ system at that stage. TFs (rows) are clustered by organ system (1 to 16) with black lines separating the organ system clusters. A TF will appear in a new row for every organ system where it is expressed. All TFs with patterned zygotic expression are included in the matrix. If a TF also shows ubiquitous staining at any stage, this is indicated by colored boxes in the five columns designated organ system 17, ubiquitous. Maternal staining (stages 1 to 3) is indicated by a single gray box with numerical code 18 in the last column of the matrix. In separate tabs, single organ system clusters are shown using numerical organ system codes without the color-coding. Click here for file

Additional file 9 **Supplemental text.** Text descriptions of TF expression profiles for all 16 organ systems, detailed legends for supplemental figures, and supplemental references. Click here for file

Additional file 10: Figure S3 SOM maps illustrating widely dispersed positions of TFs in three organ systems. Click here for file

Additional file 11: Figure S4 Developmental dynamics of TFs expressed in the visual primordia organ system. Click here for file

Additional file 12: Table S7 RNA-seq sample descriptions, accession numbers, and references. Click here for file
