# Supplementary material for: Spatial expression of transcription factors in Drosophila embryonic organ development
Source: Genome Biol. 2013 Dec 20;14(12):R140. doi: 10.1186/gb-2013-14-12-r140 (PMC4053779; doi:10.1186/gb-2013-14-12-r140)
Supplement: Additional file 1: Table S1 — InterPro, Pfam, and hand curated DNA-binding domains used to generate the TF list used in these studies. [file gb-2013-14-12-r140-S1.doc]

Table S1. InterPro, Pfam and hand curated DNA binding domains used to generate the TF list

| InterPro Accession | InterPro name | Pfam ID | Pfam name | DBD Name |
| --- | --- | --- | --- | --- |
| IPR013524 | AML1/Runt_N | PF00853 | Runt | Runt |
| IPR001606 | ARID/BRIGHT_DNA-bd | PF01388 | ARID | ARID |
| IPR017956 | AT_hook_DNA-bd_motif | PF02178 | AT_hook | AT_hook |
| IPR002546 | Basic | PF01586 | Basic | Basic |
| IPR018379 | BEN_domain | PF10523 | BEN | BEN |
| IPR004210 | BESS_motif | PF02944 | BESS | BESS |
| IPR018586 | Brinker_DNA-bd | PF09607 | BrkDBD | BrkDBD |
| IPR004827 | bZIP | none | none | bZIP |
| IPR011616 | bZIP_1 | PF00170 | bZIP_1 | bZIP_1 |
| IPR011700 | bZIP_2 | PF07716 | bZIP_2 | bZIP_2 |
| IPR003417 | CBF_beta | PF02312 | CBF_beta | CBF-beta |
| IPR003958 | CBFA_NFYB_domain | PF00808 | CBFA_NFYB_HMF | CBFA_NFYB_domain |
| IPR003957 | CBFA/NFYB_topo | none | none | CBFA/NFYB_topo |
| IPR005612 | CCAAT-binding_factor | PF03914 | CBF | CBF |
| none | none | none | none | C-clamp |
| IPR005559 | CG-1 | PF03859 | CG-1 | CG-1 |
| IPR007604 | CP2 | PF04516 | CP2 | CP2 |
| IPR004022 | DDT_domain | PF02791 | DDT | DDT |
| IPR001275 | DM_DNA-bd | PF00751 | DM | DM |
| IPR009061 | DNA-bd_dom_put | none | none | DNA_bd_put |
| IPR003316 | E2F_TDP | PF02319 | E2F_TDP | E2F_TDP |
| IPR000418 | Ets | PF00178 | Ets | Ets |
| IPR001092 | HLH_DNA-bd_dom | PF00010 | HLH | HLH |
| IPR006600 | HTH_CenpB_DNA-bd_dom | PF04218 | CENP-B_N | Cenp-B DBD1 |
| IPR000910 | HMG_HMG1/HMG2 | PF00505 | HMG_box | HMG_box |
| IPR003350 | Homeo_CUT | PF02376 | CUT | CUT |
| IPR001356 | Homeobox | PF00046 | Homeobox | Homeobox |
| IPR000232 | HSF_DNA-bd | PF00447 | HSF_DNA-bind | HSF_DNA-bind |
| IPR007889 | HTH_psq | PF05225 | HTH_psq | HTH_psq |
| IPR011526 | HTH_psq-like | none | none | HTH_psq_like |
| IPR002909 | IPT_TIG_rcpt | PF01833 | TIG | TIG |
| IPR003619 | MAD_homology1_Dwarfin-type | PF03165 | MH1 | MH1 |
| IPR006578 | MADF | PF10545 | MADF_DNA_bdg | MADF |
| IPR019526 | Nrf1_activation-bd | PF10492 | Nrf1_activ_bdg | Nrf1_DNA-bind |
| IPR014778 | Myb_DNA-bd | PF00249 | Myb_DNA-binding | Myb_DNA-binding |
| IPR025220 | NFRKB_winged | PF14465 | NFRKB | NFRKB |
| IPR011615 | p53_DNA-bd | PF00870 | P53 | P53 |
| IPR001523 | Paired_box_N | PF00292 | PAX | PAX |
| IPR000327 | POU_specific | PF00157 | Pou | Pou |
| IPR007738 | Prox1 | PF05044 | Prox1 | Prox1 |
| IPR003150 | RFX_DNA-bd | PF02257 | RFX_DNA_binding | RFX_DNA_binding |
| IPR011539 | RHD | PF00554 | RHD | RHD |
| IPR004018 | RPEL_repeat | PF02755 | RPEL | RPEL |
| IPR000770 | SAND_dom | PF01342 | SAND | SAND |
| IPR001005 | SANT_DNA-bd | none | none | SANT |
| IPR013801 | STAT_TF_DNA-bd | PF02864 | STAT_bind | STAT_bind |
| IPR000818 | TEA/ATTS | PF01285 | TEA | TEA |
| IPR007797 | TF_AF4/FMR2 | PF05110 | AF-4 | AF-4 |
| IPR013854 | TF_AP2_C | PF03299 | TF_AP-2 | TF_AP-2 |
| IPR001289 | TF_CBFB | PF02045 | CBFB_NFYA | CBFB_NFYA |
| IPR001766 | TF_fork_head | PF00250 | Fork_head | Fork_head |
| IPR002100 | TF_MADSbox | PF00319 | SRF-TF | SRF-TF |
| IPR004826 | TF_Maf | PF03131 | bZIP_Maf | bZIP_Maf |
| IPR001699 | TF_T-box | PF00907 | T-box | T-box |
| IPR003380 | Transform_Ski | PF02437 | Ski_Sno | Ski_Sno |
| IPR000580 | TSC-22 _Dip_Bun | PF01166 | TSC22 | TSC22 |
| IPR003902 | Tscrpt_reg_GCM_motif | PF03615 | GCM | GCM |
| IPR008895 | YL1 | PF05764 | YL1 | YL1 |
| IPR013272 | YL1_C | PF08265 | YL1_C | YL1_C |
| IPR002653 | Znf_A20 | PF01754 | zf-A20 | zf-A20 |
| IPR000315 | Znf_B-box | PF00643 | zf-B_box | zf-B_box |
| IPR003656 | Znf_BED_prd | PF02892 | zf-BED | zf-BED |
| IPR006612 | Znf_C2CH | PF05485 | THAP | THAP |
| IPR007087 | Znf_C2H2 | PF00096 | zf-C2H2 | zf-C2H2 |
| IPR002515 | Znf_C2HC | PF01530 | zf-C2HC | zf-C2HC |
| IPR007588 | Znf_FLYWCH | PF04500 | FLYWCH | FLYWCH |
| IPR015318 | Znf_GAGA-bd_fac | PF09237 | GAGA | GAGA |
| IPR000679 | Znf_GATA | PF00320 | GATA | GATA |
| IPR001628 | Znf_hrmn_rcpt | PF00105 | zf-C4 | zf-C4 |
| IPR004181 | Znf_MIZ | PF02891 | zf-MIZ | zf-MIZ |
| IPR000967 | Znf_NFX1 | PF01422 | zf-NF-X1 | zf-NF-X1 |
| IPR004198 | Znf-C5HC2 | PF02928 | zf-C5HC2 | zf-C5HC2 |
| none | none | none | none | zeste |
