# Supplementary figures and images for: Spatial expression of transcription factors in Drosophila embryonic organ development
Source: Genome Biol. 2013 Dec 20;14(12):R140. doi: 10.1186/gb-2013-14-12-r140 (PMC4053779; doi:10.1186/gb-2013-14-12-r140)

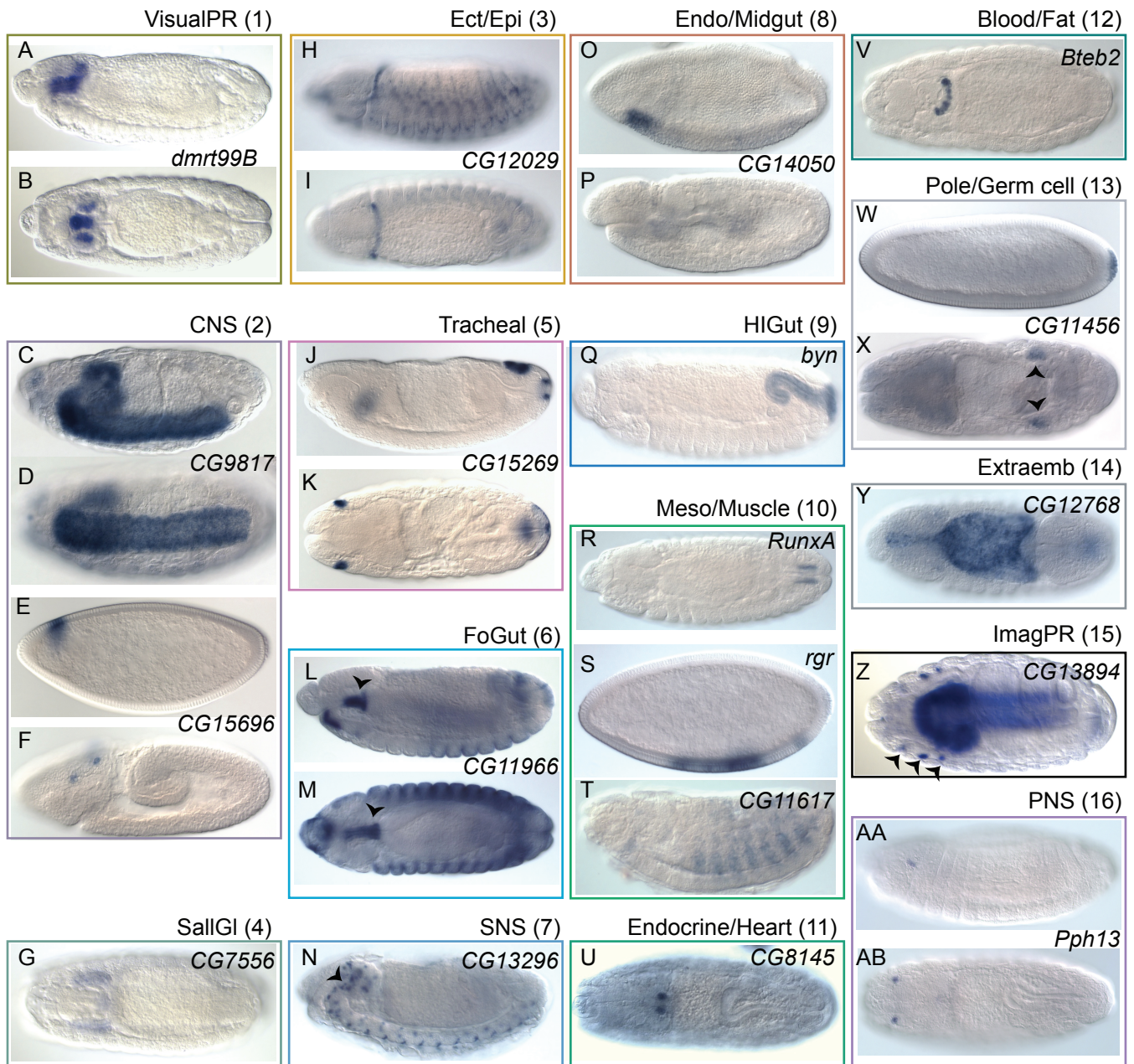

Supplement: Additional file 4: Figure S1 — Examples of TF expression patterns representing the 16 organ systems. [file gb-2013-14-12-r140-S4.pdf]

Developmental Time Course

Larval and Adult Dissected Speciment

Embryos      Larvae      Pupae      Adults

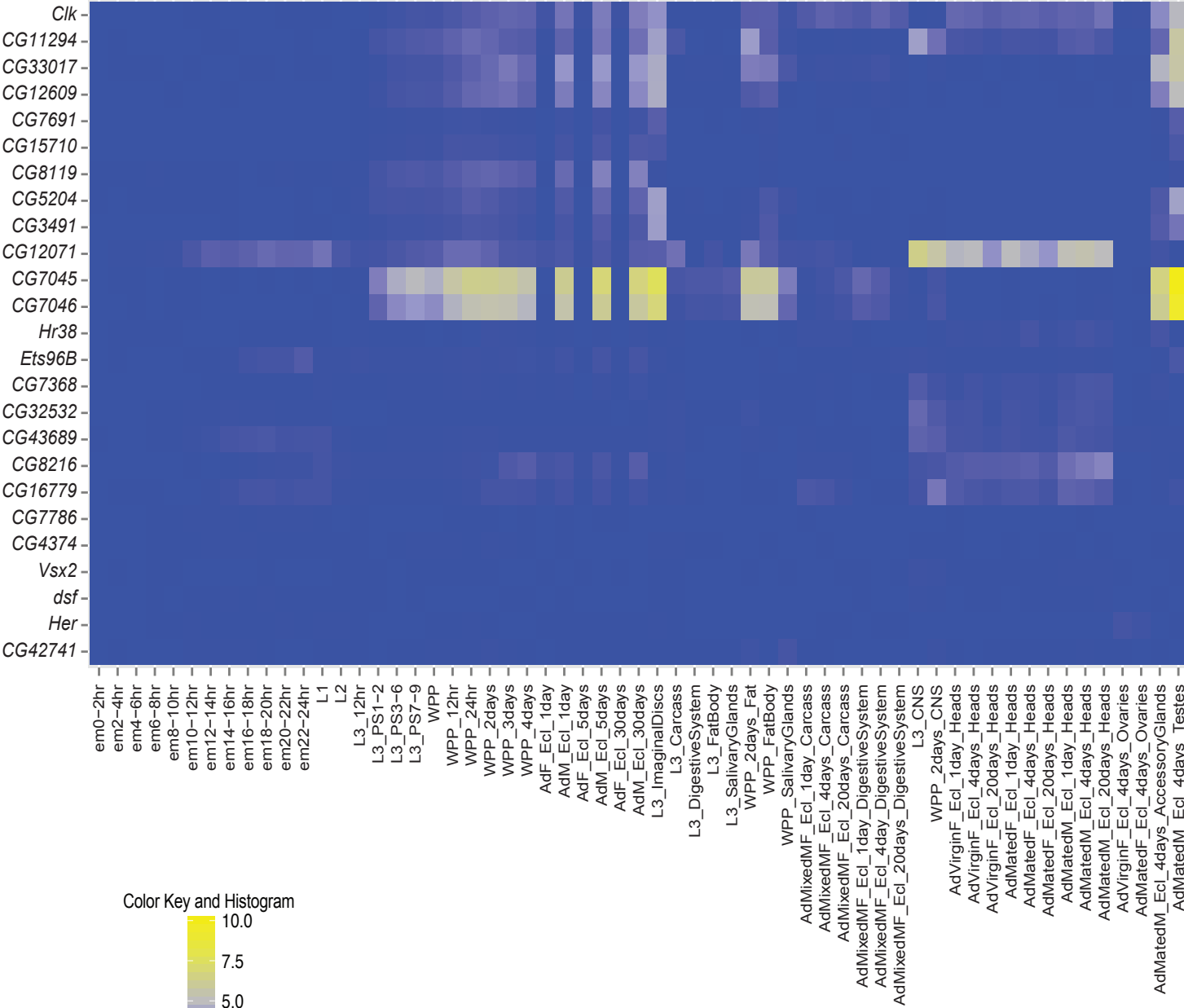

Supplement: Additional file 7: Figure S2 — Gene expression profiling of 25 TFs not detected spatially in embryos. [file gb-2013-14-12-r140-S7.pdf]

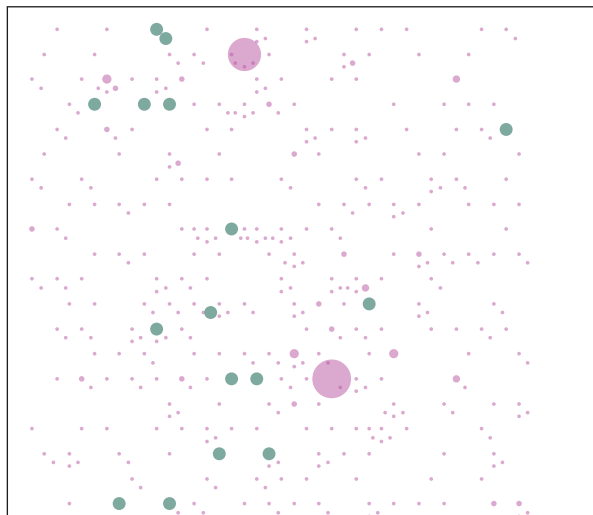

Salivary Gland 13-16

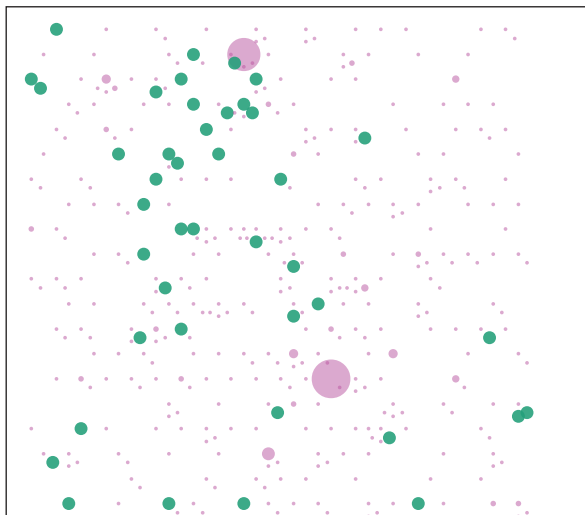

Endocrine\_Heart 13-16

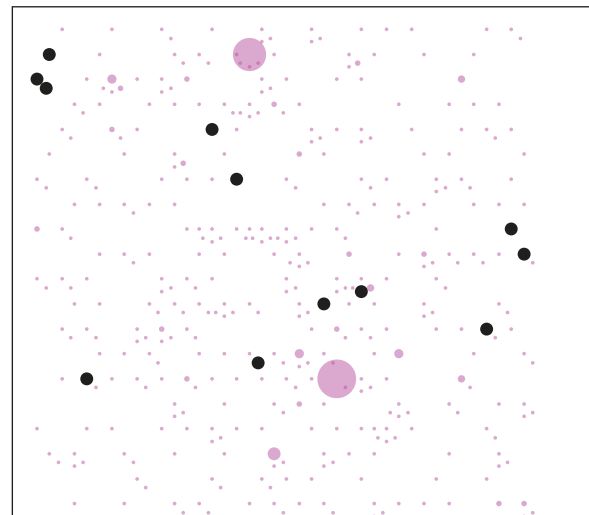

ImaginalPr 13-16

Supplement: Additional file 10: Figure S3 — SOM maps illustrating widely dispersed positions of TFs in three organ systems. [file gb-2013-14-12-r140-S10.pdf]

A

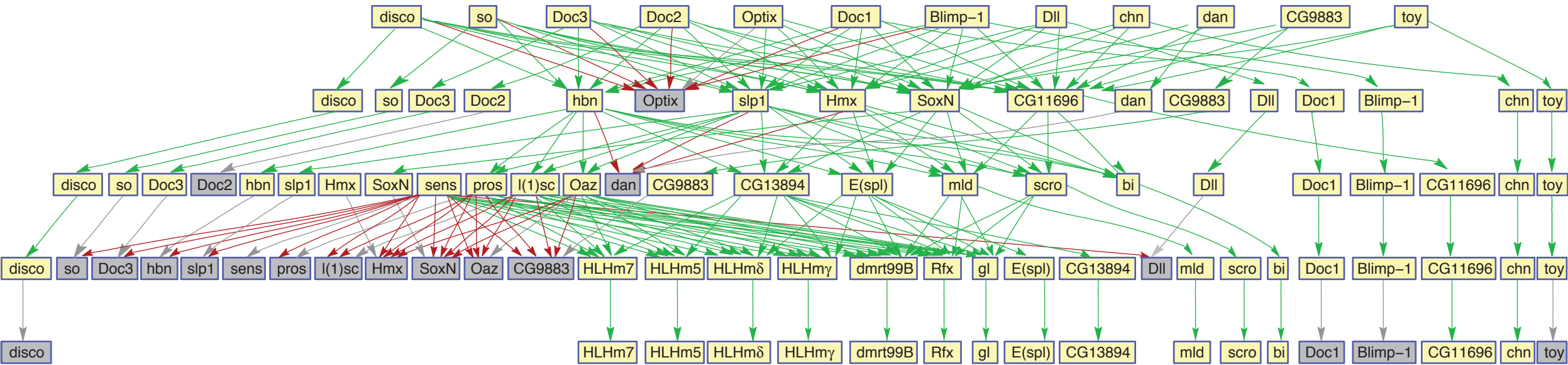

B

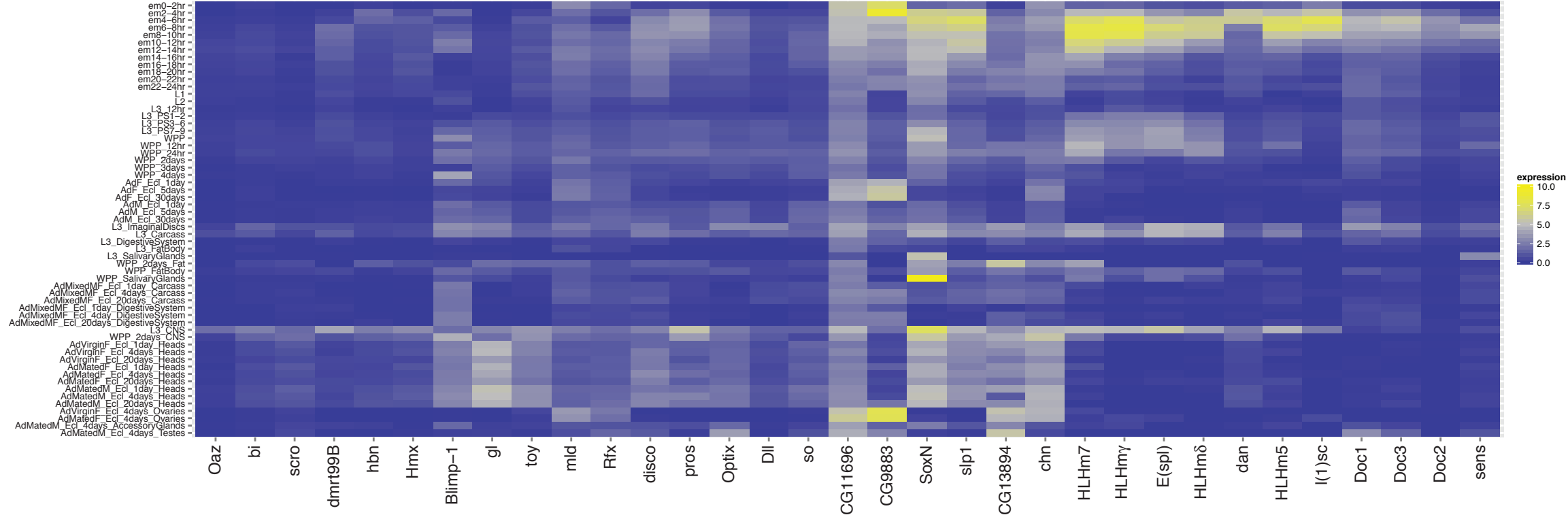

Supplement: Additional file 11: Figure S4 — Developmental dynamics of TFs expressed in the visual primordia organ system. [file gb-2013-14-12-r140-S11.pdf]
