# Supplementary material for: Spatial expression of transcription factors in Drosophila embryonic organ development
Source: Genome Biol. 2013 Dec 20;14(12):R140. doi: 10.1186/gb-2013-14-12-r140 (PMC4053779; doi:10.1186/gb-2013-14-12-r140)
Supplement: Additional file 9 — Supplemental text. Text descriptions of TF expression profiles for all 16 organ systems, detailed legends for supplemental figures, and supplemental references. [file gb-2013-14-12-r140-S9.doc]

# Additional data file 9: Supplemental Text

## TF expression summaries for 16 organ systems

The organ system (OS) clusters identified in the TF expression matrix (Additional data file 8, Table S6) provide TF expression profiles across developmental time for each of the 16 OS. Summaries of the profiles for each organ system, derived from the expression matrix are presented below. The OS numbers 1-16 refer to the organ system code numbers used in the matrix. See Additional data file 8 for further details.

*Visual Primordia (OS 1, VisualPr):* We identified 33 TFs that are expressed in the visual system primordia organ system. The visual primordia include the anlage of the optic lobe, and the primordium of the adult eye, which is integral with the head epidermis of the early embryo and invaginates as part of the eye-antennal imaginal disc during head involution in the late embryo. No TFs are restricted to the visual primordia. Nearly all TFs expressed in the visual system are expressed concurrently in the Ectoderm/Epidermis (Ect/Epi), or the CNS, or both, consistent with the ectodermal origin of these tissues. One exception is the well-characterized TF *glass (gl)* [1], which is required for normal photoreceptor development during metamorphosis [2]; in the embryo *gl* is expressed in the optic lobe anlage, in Bolwig’s organ, part of the PNS (PNS_Photo) and in the corpus cardiacum, part of the Endocrine/Heart system. Ten other TFs are expressed in both the VisualPr and the PNS. Four TFs, expressed in the VisualPr were uncharacterized previously (*CG9883, CG11696, CG13894 and dmrt99B)*. Figure S4 in Additional data file 11 shows positive and negative associations of TFs expressed in the VisualPr across developmental time. One, *CG9883,* is expressed in the anlage of the VisualPr early in embryonic development (stages 4-6). There are eleven known visual system TFs that also come on early in embryonic development. The other three are expressed in the optic lobe primordia of the terminally differentiated embryo.

*Central Nervous System (OS 2, CNS):* The CNS includes all parts of the brain and ventral nerve cord along with associated neuroblasts and glia. More than half of all TFs (362) show expression in the CNS during at least one embryonic stage. Of these, 93 TFs are expressed exclusively in the CNS, either early, defined as stages 4-8 (12 TFs), or late, defined as stages 9-16 (79 TFs), or both (2 TFs). The remaining CNS TFs are also expressed in other ectoderm-derived tissues, including epidermis, visual primordia, hindgut, foregut, and peripheral nervous system. We discovered 46 previously uncharacterized TFs that are expressed in the embryonic CNS, as well as 87 other TFs with no previous characterization of the embryonic CNS spatial expression. *CG9817* (zf-C2H2), *CG4328* (Homeobox), and *CG11093* (Ski-Sno domain), are expressed in the brain and ventral nerve cord, and RNA-seq shows that they continue to be expressed in CNS during larval, pupal and adult stages. *CG15696* and *CG32105* are two uncharacterized homeobox TFs that both show CNS expression starting at the cellular blastoderm stage. *CG32105* continues to be expressed in the developing brain and ventral nerve cord throughout embryonic development, and RNA-seq shows that it continues to be expressed in CNS during larval, pupal and adult stages. *CG15696* is expressed in the procephalic ectoderm anlage early, and its expression resolves to specific procephalic neuroblast primordia, but it is not detected by *in situ* or by RNA-seq after stage 12 {Graveley, 2011 #28}.

*Ectoderm/Epidermis (OS 3, Ect/Epi):* During gastrulation three germ layers are formed. The ectoderm is the outermost germ layer of the embryo, followed by the mesoderm and the endoderm. Derived from the ectoderm, are the epidermis, the outer epithelial layer of the embryo, the atrium or mouth cavity, the oenocytes, non-neural secretory cells, and the anal pads, involved in regulating homeostasis. Also derived from the ectoderm and described as separate organ systems are the central nervous system (OS 2), foregut (OS 6) and hindgut (OS 9). Early in embryogenesis (stages 4-10) ectodermal expression is annotated as to its dorsal or ventral location; 222 TFs are expressed in the ectoderm: 184 previously known, 13 uncharacterized and newly described here, and 25, previously characterized but not with a role in the development of the ectoderm or their derivatives. Of the newly characterized TFs, five are expressed early in the ectoderm, six have epidermal expression, either dorsal and ventral (3), dorsal only (2) or head (1) and one, *CG9876,* is expressed in the oenocytes. Four TFs were known to be expressed in developing oenocytes, *seven up* (*svp*), *pointed* (*pnt*), *spalt-related* (*salr*) and *ventral veins lacking* (*vvl*) which produce proteins of the nuclear receptor, ETS-domain, zinc-finger and POU- homeodomain class, respectively (Mlodzik et al., 1990; Scholz et al., 1993; Kuhnlein et al., 1994; Anderson et al., 1995). In addition we find *D*, *Hnf4*, *Mes2* and *sna* expressed in oenocytes.

*Salivary Gland (OS 4 SalGl:* The salivary gland organ system, composed of the salivary gland body, salivary ducts and common duct, is derived from the ventral ectoderm primordium. The salivary gland placode is first detectable at Stage 9-10. Of 28 TFs expressed in the salivary gland, only two are expressed exclusively in the salivary gland: the well-characterized salivary-specific HLH domain TF *sage* [3-5] and a previously uncharacterized SANT domain TF, *CG7556*. In addition to its restricted zygotic expression in the salivary gland, *CG7556* is maternally deposited early and expressed in post-embryonic stages in a number of tissues.

*Tracheal system (OS 5 Tracheal):* The anlage of the trachea splits off from the dorsal ectoderm at stage 9-10, forming the segmentally repeated tracheal pits and posterior spiracles. Tracheal trunks and branches begin to form at stage 11-12. We find 49 TFs expressed in the tracheal organ system. No TFs are expressed exclusively in the tracheal system, although the previously characterized bZip TF, *slow border cells* (*slbo*) [6], is restricted to the posterior spiracles and their specific anlage until stage 14, after which there is a spike of strong expression in multiple tissues at the end of embryogenesis. We discovered tracheal expression of the previously uncharacterized TF, *CG15269*  (zf-C2H2), restricted to the anterior and posterior spiracles and the anal pads, which derive from the ventral epidermis. After early ubiquitous expression that resolves to procephalic ectoderm by stage 7, the uncharacterized homeobox TF *E5* shows predominately tracheal system expression from stage 9 onward, with additional restricted expression in the head sensory system (PNS), a derivative of the procephalic ectoderm. FlyAtlas (flyatlas.org) CHiP data detect significantly enriched expression of *E5* also in the larval trachea [7].

*Foregut (OS 6 FoGut):* The foregut organ system is derived from ectoderm and consists of the anterior part of the digestive tract, including the proventriculus, esophagus, and the anlagen of somatogastric nervous system (SNS). The anlage of the foregut first appears at the blastoderm stage. We detect 152 TFs expressed in the foregut organ system. Four TFs (*caup*, *Lim1*, *scro,* and *CG12104*) show expression restricted to the foregut in early embryogenesis (stages 4-8). TFs expressed in the foregut OS often are expressed concurrently in CNS, epidermis, or hindgut, or endoderm-derived midgut. At the earliest stages, the foregut is not readily distinguishable from the anterior part of the anterior endoderm (Endo/Midgut organ system). One previously uncharacterized TF, *CG8388*, which has both an AT-hook and a zf-C2H2 domain, is expressed exclusively in the foregut and anterior endoderm anlagen until stage 10 when the expression resolves to the foregut before disappears by stage 11. Six TFs (*OdsH*, *sr*, *CG9793*, *CG8089*, *Ets98B* and *vri*) are expressed only late in embryonic development and predominantly in the foregut and epidermis.

*Stomatogastric nervous system (OS 7 SNS):* The SNS is derived from the foregut as a neuroectodermal placode, which becomes morphologically distinct starting at stage 9-10 and subsequently compartmentalizes into three pouches that develop into the SNS ganglia late in embryonic development. Over the three stage ranges of SNS development, we find expression of only 26 TFs in this organ system. SNS expression for twelve of these, including the well-characterized TFs *forkhead* (*fkd*) and *Krüppel* (*Kr*), is preceded by expression in the foregut, consistent with the foregut origin of the SNS. We find SNS expression of TFs known to be important for SNS development such as the proneural TFs *ac*, *sc* and *l(1)sc* and neurogenic TFs, *HLHmδ* and *HLHmγ*; [8, 9]. In addition, we discovered five genes with expression in the SNS that had not been previously described: *CG13296* (zf-C2H2), *CG11696* (AT_Hook, zf-C2H2), *onecut* (CUT and Homeobox domains), *dmrt99B* (DM), and *dmrt93B* (DM). The *dmrt93B* expression in the SNS is restricted to the frontal ganglion at S14-16, and it is the only TF whose expression is restricted to the SNS.

*Endoderm and midgut (OS 8 Endo/Midgut):* The anlagen of the anterior and posterior endoderm can be detected starting in the poles of the blastoderm embryo. Of the 183 TF genes expressed in the Endo/Midgut OS, only eleven are expressed exclusively in the midgut and another seven are restricted to the endoderm early but expand to additional organ systems later in embryogenesis. Most of the TFs expressed in the Endo/Midgut have broad expression patterns, including more than a third with ubiquitous expression concurrent with stronger expression in the endoderm or midgut. Among the TFs expressed in this OS are 17 previously uncharacterized TFs including CG7056, restricted to the in the posterior midgut primordium and midgut chamber starting at Stage 11, and CG14050 restricted to the endoderm and mesoderm from blastoderm through Stage 12.

*Hindgut (HiGut ) OS 9:* The hindgut is of ectodermal origin, includes the Malpighian tubules, which form close to the junction between hindgut and posterior midgut, and is subdivided into three major domains, the small intestine, large intestine, and rectum [10]. Of the 113 TFs expressed in the hindgut, many have broad expression patterns (expressed in multiple tissues), and 27 show concurrent ubiquitous and hindgut expression. One well-characterized TF, (*byn*), is expressed exclusively in the hindgut throughout embryogenesis (Figure 6). Only 25 other TFs were previously known to have roles in hindgut formation, and the remaining 60 TFs with patterned expression including hindgut expression have not been studied in in hindgut development.

*Mesoderm and muscle (Meso/Muscle) OS 10:* This is one of the most well studied organ system in flies (reviewed in [11]) with 154 TFs showing expression in a variety of patterns from early mesoderm to terminally differentiated muscles. Seventy-eight mesoderm TFs are well characterized with over 10,000 publications. Another 40 TFs are expressed ubiquitously yet are enriched in their mesodermal expression. We discovered and describe here 15 previously uncharacterized TFs, eight with expression in the mesoderm and seven with expression in the mesoderm that resolves to visceral or somatic muscle. Finally, for 34 characterized TFs, we discovered previously unreported expression in the mesoderm or muscle system.

*Endocrine and heart (OS 11 Endocrine/Heart)*: We observe 47 TFs expressed in the Endocrine/Heart organ system. Of the 47, 16 are expressed in the endocrine system, including one, *apontic* *(apt)*, which is also expressed in the heart [12, 13]. The embryonic endocrine system includes the ring gland (RG) and its components, the prothoracic gland (PG), the corpus allata (CA) and the corpus cardiacum (CC), a neuroendocrine gland. Eight TFs were previously identified by genetic and expression analysis as being required for proper CC development and seven others were known to be expressed in the endocrine system. We discovered nine TFs, *CG11762*, *CG8145,* *CG11723, CG9876, knot (kn),* *diminutive (dm), pou domain motif 3 (pdm3), Mes2 and ventral veins lacking (vv)l* with endocrine expression*.* The four CGs have not been previously characterized and are uniquely expressed in the ring gland. The five known genes were not previously characterized as having expression or function in the endocrine system.

At least twenty-three transcription factors are known to be required for proper cardiogenesis (reviewed in [14, 15]). These TFs have been identified primarily using a candidate gene approach and their roles characterized genetically. We discovered seven additional TFs with expression in cardiac tissues: *ribbon (rib), pointed (pnt), dysfusion (dys) Helix loop helix protein 106 (HLH106), drumstick (drm), Retinal Homeobox (Rx)* and *NK7.1*.

*Blood cells and fat body (Blood/Fat) OS 12.* The head mesdoderm is the primordium for two types of hemocytes, embryonic hemocytes (EH) and lymph gland hemocytes (LGH), the garland cells, a type of nephrocyte, and the fat body required for energy storage and release. As a consequence of their common descent they have been grouped together.

Both EH and LGH differentiate into podocytes, crystal cells and plasmatocytes [16]. Our understanding of the regulation of hemocyte proliferation and specification comes from genetic analysis (reviewed in [17]). Nine TFs are expressed in the blood cells: four previously known *lozenge (lz) glial cell missing (gcm), gcm2* and *serpent (srp)*, one uncharacterized and newly described here, *CG110711,* and four, Hsf, kay, slp1,and vri, previously characterized but not with a role in the development of the embryonic blood cells.

The garland cells surround the esophagus, filter haemolymph and are analogous to the vertebrate glomerulus podoctyes of the kidney [18]. The TFs that control development of the garland cells have not been identified. We discovered seven TFs, *Bteb2*, *forkhead box, sub-group O (foxo),* *Mothers against dpp (Mad)*, *MTF-1*, *Usf*, *u-shaped (ush)* and *srp* that are expressed in this tissue. Genetic analysis will reveal the structure of the regulatory network.

Little is known about the development of the embryonic fat body. Fifteen TFs are expressed in the fat body: four previously known *Dif, srp, svp* and *Pdp1*, four uncharacterized and newly described here, *CG30431, CG34376, CG8145, CG9932* and seven, *brk*, *dm*, *gem*, *kn*, *odd*, *Rel* and *zfh1* previously characterized but not known to be expressed in the embryonic fat body.

*Pole cells and germ cells (Pole/Germ cell) OS 13*): This OS includes the pole cells of the early embryo, which become the germ cells, and the gonad, formed by the joining of the germ cells with mesodermally derived somatic cells at stage 13. Although the PoleCell/GermCell OS is the only OS where we find no enrichment of expression of TFs compared to other genes (Figure 2), 49 TFs are expressed in this OS. Of these 17 are expressed exclusively in the OS either early (stages 4-8) or late (stages 9-16) Nearly half (24) of the TFs expressed in the gonad are concurrently expressed in the CNS. Fifteen of the 49 were uncharacterized previously, and another 13 were not known to be expressed or have a role in this organ system.

*Extraembryonic (Extraemb) OS 14*: The extraembryonic tissues consist of the yolk sac and the amniosera. The yolk sac is polyploid, contains yolk proteins and supplies nutritive reserves for the developing embryo. The amnioserosa performs a variety of movements as simple epithelial cell sheets, such as folding, fusion, and rupture. Both extraembryonic tissues contribute to the embryonic process, dorsal closure, which is the last major morphogenetic movement whereby the dorsal epidermis encloses the embryo and the extraembryonic membranes and yolk sac undergo apoptosis (reviewed in[19]). Forty TFs are expressed in the yolk (11) or amnioserosa (25) or both (4): 26 previously known, six uncharacterized and newly described here, and eight, previously characterized but not with a role in the development of the extraembryonic tissues.

*Imaginal primordia (ImagPR) OS 15*: Imaginal discs segregate from the larval tissue primordia in the embryo as invaginations of the embryonic epidermis, [reviewed in [20]; [21]], increase in size by cell division through the larval stages, and differentiate during metamorphosis giving rise to the adult integument. We detect gene expression in the dorsal (wing and haltere disc) and ventral (leg disc) imaginal primordia by stage 11 and in the adult eye primordium, the eye-antennal disc by stage 13. Twelve TFs are expressed non-exclusively in the imaginal primordia, with additional expression most commonly in the CNS and Ectoderm/Epidermis. Besides the well known imaginal disc embryonic markers, *snail (sna),* *escargot (esg)* and *Distal-less (Dll) eyeless (ey)* and *apterous (ap)*, we discovered *CG13894* (THAP domain) a previously uncharacterized gene expressed in the dorsal and ventral imaginal primordia from stage 13 onward. *CG13894* is expressed in the anlage of the CNS and epidermis starting in the blastoderm. RNA-seq expression studies from dissected tissues detect continued expression of *CG13894* in the larval imaginal discs and in larval and pupal CNS [Brown, 2013]. RNA-seq profiling of the wing disc derived cell line ML-DmD3 also shows *CG13894* expression (modENCODE unpublished). We discovered six other TFs, *little imaginal discs (lid*), *Dorsocross* genes (*Doc1, Doc2* and *Doc3*), *Mes2,* and *caupolican (caup)* with specific expression in imaginal precursors. The gene *lid,* named for its larval mutant phenotype [22], contains both ARID and C5HC2 zinc finger DNA binding domains as well as trimethyl H3K4 demethylase functional domains. We discovered *lid* expression in the ventral imaginal disc primoridia. The *Doc1, Doc2* and *Doc3* genes, well studied for their roles in epidermal patterning in both embryos and imaginal discs, and in amnioserosa and cardiac development [14, 23] are expressed also in the adult eye primoridium.

*Peripheral nervous system (PNS) OS 16:* The PNS is derived from the ectoderm. Of the 46 TFs sharing expression with the ectoderm, 11 TFs are expressed in the ectoderm before being restricted to the PNS, and 35 genes are expressed both in ectoderm and PNS. All but 9 TFs are shared with the CNS. Four TFs, *cato*, *Php13*, *Sox15*, and *CG32006*, are specifically restricted to the PNS. Of those, *CG32006* (Figure 1B*)* has not been described before. We found a total of eleven TFs with previously undescribed PNS expression. The new PNS TFs appear panneural with the exception of *CG16815*, which appears early during both PNS and CNS development. Few TFs are shared with the tracheal, germ cell and extraembryonic organ systems. HLH TFs have a fundamental role for specifying the PNS and CNS. Only previously described proneural and panneural TFs with a HLH domain were found expressed in this OS.

# SUPPLEMENTAL FIGURE LEGENDS

## Figure S1

Representative spatial expression patterns for each organ system. For each organ system at least one representative TF embryonic gene expression pattern is shown. A number of TFs are expressed in multiple organ systems but for this figure we focus on the organ specific expression, primarily highlighting spatial expression patterns detected here for the first time. (A-B) Visual Primordia (VisPR) features lateral and dorsal views, respectively (Stage 13) of *doublesex-Mab related 99B (dmrt99B)* with expression in the optic lobe. (C-F) Central Nervous System (CNS) features lateral and ventral views, (C-D) respectively (Stage 14) of *CG9817* with expression in the ventral nerve cord and brain. (E-F) features lateral views of *CG15696* with expression in the procephalic ectoderm and neuroblasts of Stages 5 and 11, respectively. (G) Salivary Gland (SalGl) features a ventral view of *CG7556* (Stage 13) expressed only in the salivary gland. (H-I) Ectoderm/Epidermis (Ect/Epi) features lateral and dorsal views, respectively (Stage 13) of *CG12029* with expression in the dorsal ridge and epidermis. (J-K) Tracheal System (Tracheal) features lateral and dorsal views, respectively (Stage 13) of *CG15269* with expression in the anterior and posterior spiracles. (L-M) Foregut (FoGut) features lateral and dorsal views, respectively (Stage 13) of *CG11966* with expression in the foregut (arrowheads). (N) Stomatogastric nervous system (SNS) features a lateral view, (Stage 13) of *CG13296* with expression in the stomatogastric nervous system (arrowhead), ventral nervous system and brain (glial cells). (O-P) Endoderm/Midgut (Endo/Midgut) features lateral views of *CG14050* at Stage 6 with expression in the anterior endoderm and at Stage 11 with expression in the anterior and posterior midgut primordia. (Q) Hindgut (HiGut) features a lateral view (Stage 13) of the previously described gene, *brachyenteron (byn)* with expression in the hindgut and anal pads [24]. (R-T) Mesoderm/Muscle (Meso/Muscle) features three genes, *CG11966, regular (rgr* ) and *CG11617*. *CG11966* is expressed in the visceral mesoderm surrounding the posterior hindgut (dorsal view Stage 13). *rgr* is expressed in the trunk mesoderm (lateral view Stage 5)*. CG11617* is expressed in the somatic muscle (lateral view Stage 13). (U) Endocrine/Heart features a dorsal view (Stage 13) of *CG8145* with expression in the ring gland, part of the endocrine system. (V) Blood/Fat features a dorsal view (Stage 13) of *Bteb2* with expression in the garland cells that take up waste products from the haemolymph. (W-X) Pole/Germ cell features dorsal views of *CG11456* at Stage 6 showing expression in the pole cells and at Stage 13, respectively, showing expression in the gonads (arrowheads). (Y) Extraembryonic (Extraemb) features a dorsal view, (Stage 13) of *CG12768* with expression in the amnioserosa. (Z) Imaginal Primordia (ImaginalPR) features a ventral view, (Stage 13) of *CG13894* with expression in the dorsal imaginal precursors (arrowheads). (AA-AB) Peripheral Nervous System (PNS) features lateral and dorsal views, respectively (Stage 13) of *PvuII-PstI homology 13 (Pph13)* with expression in the cells of the Bolwig’s organ.

## Figure S2

RNA-seq expression of TFs with no detectable embryonic spatial expression. Heat map showing TF expression in 30 developmental stages and 29 larval and adult dissected specimens. Of the 25 TFs with no detectable embryonic spatial expression pattern, 24 have RNA-seq expression < 1 RPKM in the embryonic stages before cuticle deposition. *CG12071,* the only exception, has RNA-seq embryonic expression scores > 1 RPKM. Based on its high RNA-seq expression scores in L3 and WPP_2day CNS and adult head samples, it is likely to be expressed in a few embryonic neural cells that were missed in our experiments. Of the 25 TFs, 18 are clearly expressed in later stages of development: eleven, *Ets96B*, *Clk, CG7691, CG7045, CG7046, CG5204, CG3491, CG33017, CG15710, CG12609* and *CG11294* start to be detectable in late larval and pupal stages and continue to be expressed in 1, 5 and 30 day males primarily though not exclusively in dissected accessory glands and testes, the other seven, *Hr38, CG8216, CG7368, CG43689, CG32532, CG16779* and *CG12071* are primarily detectable in the dissected adult head samples. Expression of CG8119 is detected in late larvae, pupae and adults but not in our dissected tissue samples. Finally, six*, Vsx2, Her, dsf, CG42741, CG7786* and *CG4374*, are very lowly expressed. Of these three are known to be weakly expressed at late stages of development, *Vsx2 (*a few cells in the medulla cortex region of L3 larval brains [25], *Her* (low expression in the adult ovaries [7]) and *dsf* (“extremely limited set of neurons in regions of the brain potentially involved in sexual behavior” [26].

## Figure S3

SOM map positions for TFs expressed in the SalGl, Endocrine/Heart, and ImagPr organ systems. The three maps are as described for Figure 5. Colored dots overlay map positions for the TFs expressed in each of these three organ systems at stage 13-16, which show more widely dispersed distribution on the maps compared to the TF distributions of the organ system shown in Figure 5.

## Figure S4

Developmental dynamics of TFs expressed in the Visual Primordia Organ System. (A) Five rows show genes expressed (yellow boxes) or inactivated (gray boxes) at five zygotic stage ranges, 4-6 (1:20-3 hrs; visual *anlage in statu nascendi* (AISN)), 7-8 (3-3:40 hrs; visual anlage), 9-10 (3;40-5:20 hrs; visual primordia), 11-12 (5:20-9:20 hrs; visual primordia 2, optic lobe primordia, inner optic lobe primordia, outer optic lobe primordial, adult eye primordia ), and 13-16 (9:20-16 hrs; visual system, optic lobe, inner optic lobe, outer optic lobe, adult eye) captured by the in situ annotations, shown sequentially from the top row. Green arrows indicate TFs that are positively associated with expression of another TF in the next developmental stage and red arrows indicate TFs that are negatively associated with the expression of another TF in the following stage. Gray arrows show TFs with negative association with their own expression in the following stage. (B) RNA-seq visualized as a heat map showing expression of the same TFs in (A) in 30 developmental stages and 29 larval and adult dissected specimens.

# Supplemental References

1. De Velasco B, Shen J, Go S, Hartenstein V: **Embryonic development of the Drosophila corpus cardiacum, a neuroendocrine gland with similarity to the vertebrate pituitary, is controlled by sine oculis and glass.** *Dev Biol* 2004, **274:**280-294.

2. Moses K, Rubin GM: **Glass encodes a site-specific DNA-binding protein that is regulated in response to positional signals in the developing Drosophila eye.** *Genes Dev* 1991, **5:**583-593.

3. Moore AW, Barbel S, Jan LY, Jan YN: **A genomewide survey of basic helix-loop-helix factors in Drosophila.** *Proc Natl Acad Sci U S A* 2000, **97:**10436-10441.

4. Chandrasekaran V, Beckendorf SK: **senseless is necessary for the survival of embryonic salivary glands in Drosophila.** *Development* 2003, **130:**4719-4728.

5. Abrams EW, Mihoulides WK, Andrew DJ: **Fork head and Sage maintain a uniform and patent salivary gland lumen through regulation of two downstream target genes, PH4alphaSG1 and PH4alphaSG2.** *Development* 2006, **133:**3517-3527.

6. Rorth P, Montell DJ: **Drosophila C/EBP: a tissue-specific DNA-binding protein required for embryonic development.** *Genes Dev* 1992, **6:**2299-2311.

7. Chintapalli VR, Wang J, Dow JA: **Using FlyAtlas to identify better Drosophila melanogaster models of human disease.** *Nat Genet* 2007, **39:**715-720.

8. Hartenstein V, Tepass U, Gruszynski-deFeo E: **Proneural and neurogenic genes control specification and Morphogenesis of stomatogastric nerve cell precursors in Drosophila.** *Dev Biol* 1996, **173:**213-227.

9. Gonzalez-Gaitan M, Jackle H: **Invagination centers within the Drosophila stomatogastric nervous system anlage are positioned by Notch-mediated signaling which is spatially controlled through wingless.** *Development* 1995, **121:**2313-2325.

10. Takashima S, Murakami R: **Regulation of pattern formation in the Drosophila hindgut by wg, hh, dpp, and en.** *Mech Dev* 2001, **101:**79-90.

11. Maqbool T, Jagla K: **Genetic control of muscle development: learning from Drosophila.** *J Muscle Res Cell Motil* 2007, **28:**397-407.

12. Gellon G, Harding KW, McGinnis N, Martin MM, McGinnis W: **A genetic screen for modifiers of Deformed homeotic function identifies novel genes required for head development.** *Development* 1997, **124:**3321-3331.

13. Su MT, Venkatesh TV, Wu X, Golden K, Bodmer R: **The pioneer gene, apontic, is required for morphogenesis and function of the Drosophila heart.** *Mech Dev* 1999, **80:**125-132.

14. Reim I, Frasch M: **Genetic and genomic dissection of cardiogenesis in the Drosophila model.** *Pediatr Cardiol* 2010, **31:**325-334.

15. Monier B, Tevy MF, Perrin L, Capovilla M, Semeriva M: **Downstream of homeotic genes: in the heart of Hox function.** *Fly (Austin)* 2007, **1:**59-67.

16. Mandal L, Banerjee U, Hartenstein V: **Evidence for a fruit fly hemangioblast and similarities between lymph-gland hematopoiesis in fruit fly and mammal aorta-gonadal-mesonephros mesoderm.** *Nat Genet* 2004, **36:**1019-1023.

17. Meister M, Lagueux M: **Drosophila blood cells.** *Cell Microbiol* 2003, **5:**573-580.

18. Weavers H, Prieto-Sanchez S, Grawe F, Garcia-Lopez A, Artero R, Wilsch-Brauninger M, Ruiz-Gomez M, Skaer H, Denholm B: **The insect nephrocyte is a podocyte-like cell with a filtration slit diaphragm.** *Nature* 2009, **457:**322-326.

19. Munoz-Descalzo S, Terol J, Paricio N: **Cabut, a C2H2 zinc finger transcription factor, is required during Drosophila dorsal closure downstream of JNK signaling.** *Dev Biol* 2005, **287:**168-179.

20. Diaz-Benjumea FJ, Cohen SM: **Interaction between dorsal and ventral cells in the imaginal disc directs wing development in Drosophila.** *Cell* 1993, **75:**741-752.

21. Fristrom D, Wilcox M, Fristrom J: **The distribution of PS integrins, laminin A and F-actin during key stages in Drosophila wing development.** *Development* 1993, **117:**509-523.

22. Gildea JJ, Lopez R, Shearn A: **A screen for new trithorax group genes identified little imaginal discs, the Drosophila melanogaster homologue of human retinoblastoma binding protein 2.** *Genetics* 2000, **156:**645-663.

23. Reim I, Frasch M: **The Dorsocross T-box genes are key components of the regulatory network controlling early cardiogenesis in Drosophila.** *Development* 2005, **132:**4911-4925.

24. Kispert A, Herrmann BG, Leptin M, Reuter R: **Homologs of the mouse Brachyury gene are involved in the specification of posterior terminal structures in Drosophila, Tribolium, and Locusta.** *Genes Dev* 1994, **8:**2137-2150.

25. Erclik T, Hartenstein V, Lipshitz HD, McInnes RR: **Conserved role of the Vsx genes supports a monophyletic origin for bilaterian visual systems.** *Curr Biol* 2008, **18:**1278-1287.

26. Finley KD, Edeen PT, Foss M, Gross E, Ghbeish N, Palmer RH, Taylor BJ, McKeown M: **Dissatisfaction encodes a tailless-like nuclear receptor expressed in a subset of CNS neurons controlling Drosophila sexual behavior.** *Neuron* 1998, **21:**1363-1374.
